# An Overview on Composite Sorbents Based on Polyelectrolytes Used in Advanced Wastewater Treatment

**DOI:** 10.3390/polym13223963

**Published:** 2021-11-16

**Authors:** Florin Bucatariu, Carmen Teodosiu, Irina Morosanu, Daniela Fighir, Ramona Ciobanu, Larisa-Maria Petrila, Marcela Mihai

**Affiliations:** 1 “Petru Poni” Institute of Macromolecular Chemistry, 41A Grigore Ghica Voda Alley, 700487 Iasi, Romania; fbucatariu@icmpp.ro (F.B.); larisa.petrila@icmpp.ro (L.-M.P.); 2 Department of Environmental Engineering and Management, “Gheorghe Asachi” Technical University of Iasi, 73 D. Mangeron Street, 700050 Iasi, Romania; morosanu.irina@gmail.com (I.M.); daniela.arsene@ch.tuiasi.ro (D.F.); ramona.ciobanu@student.tuiasi.ro (R.C.)

**Keywords:** advanced wastewater treatment, polyelectrolytes, composites, sorption, heavy metal ions, inorganic pollutants, organic pollutants

## Abstract

Advanced wastewater treatment processes are required to implement wastewater reuse in agriculture or industry, the efficient removal of targeted priority and emerging organic & inorganic pollutants being compulsory (due to their eco-toxicological and human health effects, bio-accumulative, and degradation characteristics). Various processes such as membrane separations, adsorption, advanced oxidation, filtration, disinfection may be used in combination with one or more conventional treatment stages, but technical and environmental criteria are important to assess their application. Natural and synthetic polyelectrolytes combined with some inorganic materials or other organic or inorganic polymers create new materials (composites) that are currently used in sorption of toxic pollutants. The recent developments on the synthesis and characterization of composites based on polyelectrolytes, divided according to their macroscopic shape—beads, core-shell, gels, nanofibers, membranes—are discussed, and a correlation of their actual structure and properties with the adsorption mechanisms and removal efficiencies of various pollutants in aqueous media (priority and emerging pollutants or other model pollutants) are presented.

## 1. Introduction

Municipal wastewater treatment plants (MWWTPs) face great challenges in optimizing technologies to avoid ecological and human health problems and to ensure environmental sustainability, in direct correlation with the increased pollution due to economic and social growth, wastewater quality discharged into surface waters and climate changes. Industrial activities are responsible for the discharge of effluents with a wide range of inorganic and organic compounds that belong to the priority (PPs) and emerging pollutants (EPs) classes, pharmaceuticals and personal care products, pesticides, heavy metals, detergents, flame-retardants being only few examples of such pollutants. Through their presence, eco-toxicological and human health effects, bio-accumulative and degradation characteristics may influence aquatic biota and the performances and costs of water and wastewater treatment technologies [1]. Moreover, considering the new Circular Economy Action Plan and EU Green Deal there is a huge pressure nowadays on the regional water operators (running water and wastewater treatment plants) to decrease their operational costs and associated environmental impacts and carbon footprints (especially due to energy consumption), while introducing more viable alternatives for wastewater recycling for industries, agriculture/irrigation, aquacultures, trying to recover materials and energy from wastewater sludge [2,3].

**Figure 1:**
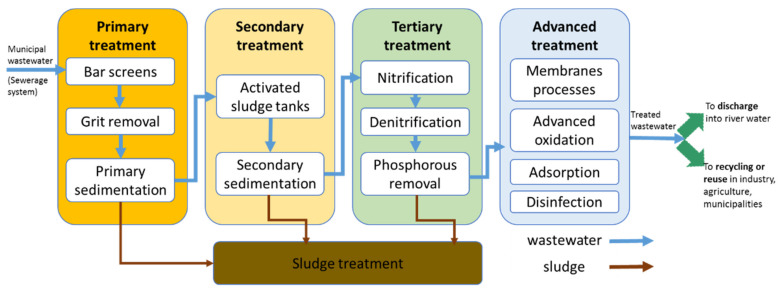
Municipal wastewater treatment outlines for various uses and pollutants removal.

MWWTPs collecting wastewater from combined sewers usually remove solids of different sizes, biodegradable organic and inorganic compounds based on conventional processes such as the following: mechanical (bar-screens, grit removal and sedimentation), biological (suspended or attached growth) and tertiary (nitrogen and phosphorous removal) treatment. The implementation of wastewater reuse in agriculture or industry requires the elimination of targeted priority and emerging organic & inorganic pollutants, microorganisms (bacteria, viruses, parasites) by means of advanced wastewater treatment (AWWT) (such as membrane processes, advanced oxidation, adsorption, filtration, disinfection, etc.), that complete the conventional treatment as presented in Figure 1.

Although, inorganic and organic pollutants classified as PPs or EPs are detected in wastewater at low concentrations (few micrograms/milligrams per liter), they are toxic, bio-accumulative, low biodegradable and very difficult to remove in terms of technological, energy and environmental efforts. In the European Union, both PPs and EPs are monitored in surface water [4,5,6], but at the moment, in Europe, only Switzerland enforces legal obligations to remove these compounds within MWWTPs [7]. The advanced wastewater treatment for EPs removal (for wastewater recycling and reuse) should consider at least the following criteria: (i) range of treated pollutants, treatment efficiency & removal mechanisms, (ii) environmental reduced impacts, (iii) simplicity of operation & maintenance, (iv) cost-effectiveness, (v) social acceptance [8].

In recent years, numerous classes of inorganic (metal oxides, zeolites, sand), organic (activated carbon, resins, covalent organic frameworks), or composite sorbents have been designed to sorb different classes of organic/inorganic pollutants [9,10,11,12,13,14,15]. Due to some disadvantages (high costs, low sorption capacities, low number of reusing cycles, non-degradable characteristics, secondary pollution) many types of these materials could be difficult to be used in practice at large scale. Recently, natural and synthetic polyelectrolytes were combined with some inorganic materials (SiO_2_, TiO_2_, graphene oxide, Fe_3_O_4_, clays, zeolites etc.) or other organic polymers (cellulose, wood, cyclodextrin, polystyrene (PS), etc.) to create new materials (composite type) as perfect candidates for sorption of toxic pollutants such as pharmaceuticals and heavy metal ions [16,17].

The main objective of this study is to present the recent development on the manufacturing of composite polyelectrolytes with sorption properties of organic and inorganic pollutants from the aqueous effluents. Specifically, this study will provide (a) an overview of the synthesis and characterization of composites based on polyelectrolytes, divided according to their macroscopic shape: beads, core-shell, gels, nanofibers, membranes; (b) correlation of their actual structure and properties with the adsorption mechanisms and removal efficiencies of various pollutants in aqueous media (PPs, Eps, or other model pollutants).

## 2. Methodology

### 2.1. Literature Search and Eligibility Criteria

In this review we exclusively focused on studies that have investigated the composite polyelectrolytes used to remove heavy metal ions and organic compounds from wastewater through sorption processes. The database selected for this research was Scopus and the search was employed from April to September 2021.

For the eligibility criteria [1] we excluded all the studies that:

- were not written in English (criteria 1);
- were not published between 2011–2021 (criteria 2);
- did not belong to the subject areas of chemistry, environmental science, chemical engineering, material science, and engineering (criteria 3);
- did not contain keywords such as beads OR core-shell OR gels OR nanofiber OR membranes (criteria 4).

### 2.2. Selections of the References

The following keywords have been used in the following combination: composite polyelectrolytes AND wastewater treatment AND sorption AND (heavy metal* OR pharmaceutical*). The search returned 517 publications, but the language of the papers was restricted to English (criteria 1), resulting in 511 documents. We used pharmaceutical compounds as model pollutants for EPs, due to their relevant interest for removal by means of advanced wastewater treatment processes.

**Figure 2:**
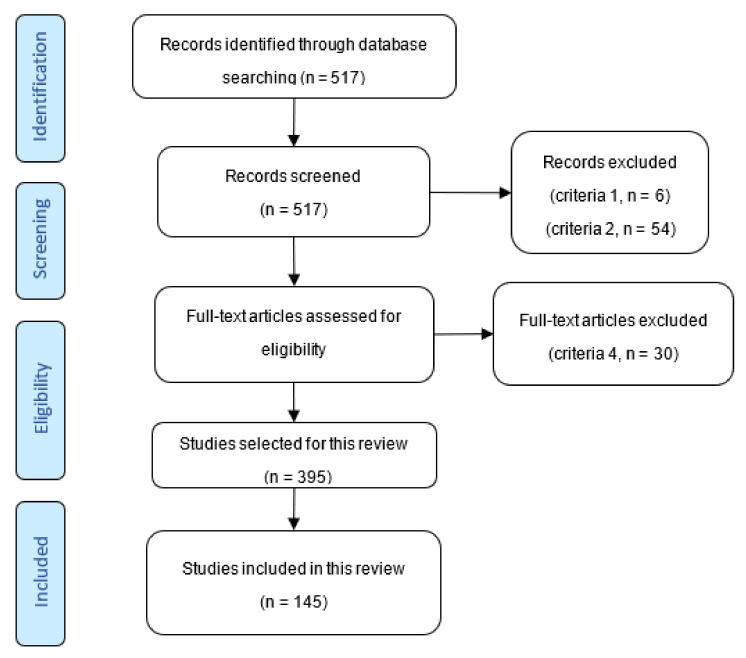
The Prisma flow diagram of the study selection process.

We selected the studies published in the period 2011–2021 (criteria 2) (Figure 2). After the elimination we obtained 457 documents. In this paper we have considered the publications that include research on the following subject areas (criteria 3): chemistry, environmental science, chemical engineering, material science and engineering (425 documents including 253 articles, 121 reviews, 38 book chapters, 11 books, and 2 conference papers).

The next selection includes other keywords related to the type of composite polyelectrolytes presented in this research: beads OR core-shell OR gels OR nanofiber OR membranes (criteria 4), only 395 relevant references were analyzed as full content through manual selection. As the reader will notice in Section 3, our manual selection resulted in the inclusion of articles presenting the adsorptive ability of composite polyelectrolytes concerning dyes. The authors decided so in line with the large use of dyes as model pollutant for the organic pollutants category. In addition, only few articles could be found reporting emerging pollutants elimination using composite polyelectrolytes.

## 3. Composite Polyelectrolytes with Versatile Properties in Targeting Different Types of Pollutants Dissolved in Real/Simulated Aqueous Effluents

### 3.1. Why Composites Based on Polyelectrolytes?

Polyelectrolytes are charged/chargeable polymers whose repeated ionic/ionizable structural units are higher than 10–15 mol%. Based on their ability to partially/fully dissociate in aqueous environment, polyelectrolytes are usually classified in weak or strong and in anionic and cationic in dependence of charge type. These polymeric compounds have a great potential in water and wastewater treatment (such as coagulation-flocculation process) due to a particular characteristic: the high densities of functional groups bound to a flexible polymeric chain, allowing the intimate interaction at the atomic level with various types of pollutants.

Using polyelectrolyte characteristics, numerous types of composites can be fabricated by covalent bonding or non-covalent bonding (hydrogen bonding, π–π stacking, metal-ligand coordination, ionic, and hydrophobic interactions) between polyelectrolytes and different inorganic/organic partners (Table 1). Composite polyelectrolyte materials, which include mainly polymeric ionic chains, contain a large number and specific stimuli-responsive functional groups with controllable action (detection, immobilization, releasing) toward different classes of pollutants entities found in aqueous environment [18,19]. The main types of polyelectrolytes, polymers, and inorganics entities used in creation of composite sorbents, as well as the main tested pollutants, have been summarized in Table 1.

The overview in Table 1 suggests the predominant use of heavy metals and dyes (HM&D) as models for inorganic and organic aqueous pollutants, respectively. From our testing experience, these pollutants present certain advantages as compared to more complex molecules, like emerging pollutants:

- chemical structure—HM&D are usually present in solution in ionic form and have smaller molecular weights, allowing them to diffuse more easily through the sorbent’s pores and reach the active sites;
- range of initial concentration during adsorption tests—HM&D are used up to hundreds of mg/L, while EPs usually are at most in the first few tens of mg/L;
- analysis equipment—HM&D can be determined by using more affordable, less time consuming and potentially less sophisticated equipment (e.g., UV-Vis spectrophotometer, atomic absorption spectrometer).

Every sorption process of the active species implies physical (transport, flow, swelling, diffusion etc.) and chemical (interactions, immobilization) aspects. First of all, the pollutant molecules, dissolved in a certain medium, must be transported near to the active site of the composite sorbent. Then, based on physico-chemical interactions between sorbate and sorbent, the sorption process can take place. Therefore, the shape and size of each composite sorbent at macro/nanoscale will dictate the active specific surface available for subsequent chemical interactions. The swelling will influence the diffusion of sorbate through the dynamic pores and the composite particle size will dictate the potential applications at laboratory or industrial scale. For example, composites larger than 10 microns could be used in a column experimental set-up.

**Figure 3:**
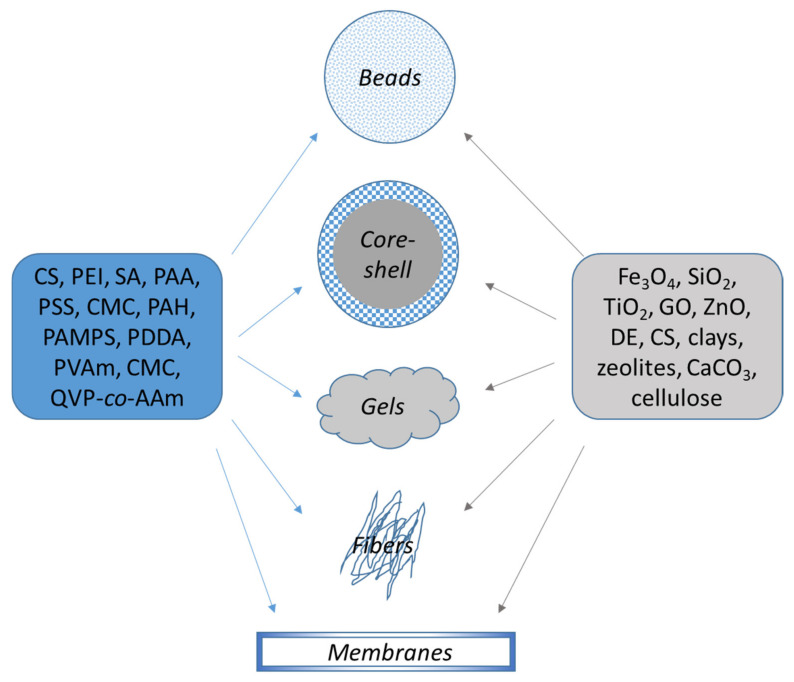
Organic/inorganic composite sorbents based on polyelectrolytes.

Thus, in this study all composites based on polyelectrolytes have been divided according to their macroscopic shape: (1) **Beads**—high porous materials with high accessibility to the functional moieties; (2) **Core-shell**, “hard-soft” composites with high surface area; (3) **Gels** (hydrogels, cryogel monoliths, sponges), stimuli-responsive systems with high surface area; (4) **Nanofibers**, presenting good mechanical properties with high specific area and (5) **Membranes**, with sorption and separation in one step (Figure 3).

The use of inorganic/organic or organic/organic composites with polyelectrolyte(s) as active part of the sorbent proved to be of high interest for immobilization of different pollutant types from aqueous environment, due to the large variety and high numbers of functional groups (carboxylic, sulphonic, amine, imine, hydroxyl) [125]. Immobilization efficiency and selectivity through complexation, ion exchange, electrostatic and hydrophobic interactions were linked to the nature of both functional groups and pollutants.

**Figure 4:**
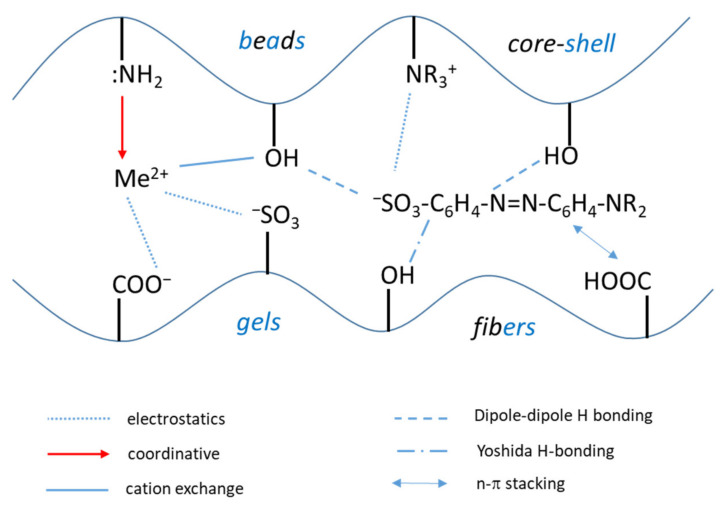
Principal types of interactions between inorganic (Me^2+^) and organic pollutants with composite polyelectrolyte sorbents functional groups.

The nature of composite components, the cross-linking density of the synthesized material and the polyelectrolyte architecture significantly influence the sorption properties and kinetics. Thus, after the physical state of composites (shape, size, density, cross-linking degree etc.), the chemical interactions will be the second important parameter that dictates the material capacity for pollutant detection, immobilization, concentration in solid phase and subsequent releasing for a new starting sorption cycle. The sorption process of inorganic (Me^2+^) or organic pollutants (dyes, pharmaceuticals etc.) are driven mainly by electrostatic interactions, coordinative bonds formation, dipole-dipole or Yoshida H-bonding interactions, and ion exchange interactions. The schematic diagram of all these interactions is presented in Figure 4.

This review brings a clear outlook on the benefits of using different moieties types as active sites of composites based polyelectrolytes for the removal by sorption mechanism of a wide range of toxic, undesired pollutants dissolved in water and wastewaters. The following subsections will be in accordance with the physical state of composite materials, such as beads, core-shells, gels, fibers and membranes, where the chemistry behind each pollutant retention is based only on sorptive mechanisms.

### 3.2. Beads Composites Based on Polyelectrolytes

In the last years, numerous studies have been focused towards combination between organic polymers and organic/inorganic entities (see Table 1) due to their structural diversity, which can be successfully used in removal of inorganic/organic pollutants dissolved in aqueous media. Sorption processes in water treatment could require granular materials with certain size and, thus, the beads/microbeads composites based on polyelectrolytes could widen the possibilities of pollutants extraction in solid phases. Novel binary/ternary beads composites, where polyelectrolyte chain is the main component, could be mainly obtained by: (a) combination of CS with organic species (e.g., starches-*g*-polyacrylonitrile [53], carrageenan [31], PVAm [54], PEI [55], microcrystalline cellulose [56], carboxymethyl-*β*-cyclodextrin [42], QCS [20,60], citrate [52]) and/or inorganic (e.g., Fe_3_O_4_ [52], Fe [42]), (b) combination of SA with organic/inorganic entities, such as activated carbon [77], CMC [79], polyaniline [61], different types of clays (bentonite [63,78,80], ilite [76], kaolinite [76], montmorillonite [68,81,82], Fe_3_O_4_ [59,64,67], SiO_2_ [65], hydroxyapatite [66]), and (c) combination of other types of polyelectrolytes (PEI, PAA) [97] to form interpolyelectrolyte composite beads. The physico-chemical integrity of combined architectures inside the bead composite must be kept under different environmental conditions (pH, ionic strength, temperature, magnetic field, etc.); therefore, the cross-linking of organic/inorganic components is very important for beads stability/integrity and functional groups subsequent accessibility by the pollutant molecule. The cross-linking could be carried out in situ (emulsification method) during the bead formation or after the coagulation/precipitation or frozen of composite beads. The most important cross-links could be achieved by covalent or ionic cross-linking with bifunctional compounds (e.g., glutaraldehyde [53,62], ECH [20,31,54,55,96], poly(ethyleneglycol diglycidyl ether) [53], carbodiimide [42,52], 3-chloro-2-hydroxypropyl trimethyl ammonium chloride [60] etc.) and small ions (ionic gelation method), respectively, including Ca^2+^ [56,61,63,64,65,66,68,76,77,78,80,82], Ce^3+^ [67], Zr^4+^ [59], Fe^3+^), tripolyphoshate [54]. Bifunctional reagents (glutaraldehyde, ECH etc.) can react with primary amino groups (CS, PEI, PVAm) to form covalent cross-links, while small ions such as Ca^2+^, Fe^3+^, or Zr^4+^ can ionically interact with two, three, or four carboxyl groups of SA.

**Figure 5:**
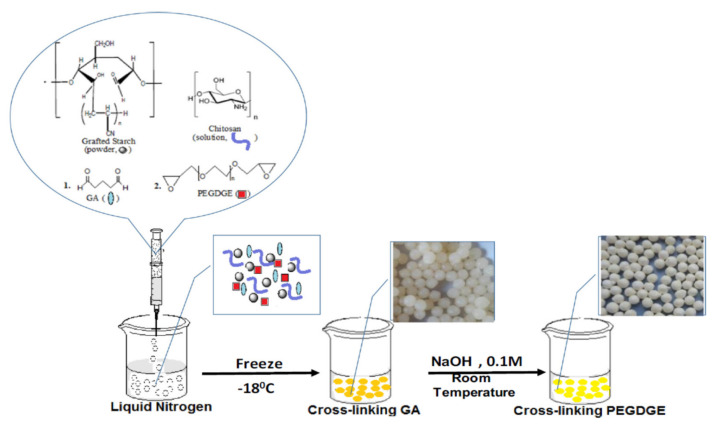
Composite cryobeads based on CS and starch (reprinted with permission from ref. [53]).

Dragan and Apopei Loghin [53] obtained biosorbents cryobeads from chitosan and starch, using glutaraldehyde and poly(ethylene glycol diglycidyl ether) as dual cross-linkers (Figure 5). It was shown that these composite cryobeads kept their integrity and sorption capacity toward three heavy metal ions (Cu^2+^, Ni^2+^ and Co^2+^) during five sorption/desorption cycles.

Numerous authors obtained composite beads with magnetic properties by embedding Fe_3_O_4_ or Fe inside the polymeric matrix, which can be CS/carrageenan [31], CS/carboxymethyl-*β*-cyclodextrin [42], carboxymethyl chitosan/chitosan/citrate [52], calcium alginate [64], cerium alginate [67] etc., feasible to be used both in batch and column sorption studies.

**Figure 6:**
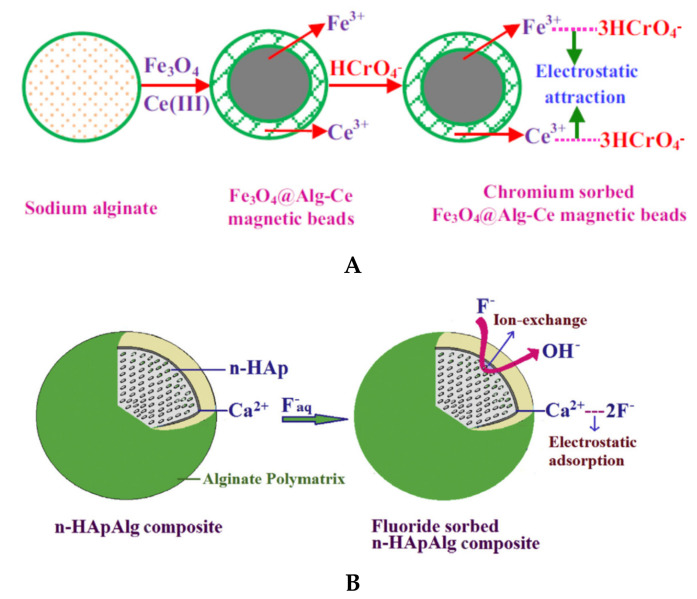
Structure of SA/magnetic (**A**) (reprinted with permission from ref. [67]) and SA/hydroxyapatite (**B**) composite beads (reprinted with permission from ref. [66]) and subsequent interaction with pollutants.

Magnetic composites beads based on natural polyelectrolytes have attracted the scientists’ attention due to the high sorption/selectivity capacity. Liang and co-workers [31] obtained composite beads with magnetic responsiveness, showing high sorption efficiency toward dyes (MB and CR) and heavy metal ions (Cu^2+^ and Cr^3+^). Gopalakannan and Viswanathan [67] obtained magnetic alginate composites beads by incorporating Fe_3_O_4_ into SA network followed by Ce^3+^ ionic gelation (Figure 6). The synthesized beads presented higher sorption capacity for chromate ions (14.29 mg/g) compared with beads without Fe_3_O_4_ (9.45 mg/g) and single Fe_3_O_4_ particles (9.72 mg/g). In this study it was shown the spontaneous and endothermic nature of chromium sorption.

To improve the mechanical stability and porosity/accessibility inside the composite beads, different types of clays (bentonite, kaolinite, montmorillonite), SiO_2_, hydroxyapatite etc., have been included during the beads synthesis [63,65,66,68,76,80], where the inorganic component could act as a cross-linker agent together with small ions, such as Ca^2+^. Pandi and Viswanathan [66] showed that defluorination capacity of hydroxyapatite (1296 mg F^−^/kg) and SA beads (680 mg F^−^/kg) increased to 3870 mg F^−^/kg for SA/hydroxyapatite composite beads. Belhouchat and co-workers [63] used bentonite to obtain SA/bentonite composite beads with high sorption capacity for anionic dyes, such as MB and MO. The authors observed that both anionic and cationic dyes could be immobilized by SA composite beads with bentonite inside. The MO sorption increased with bentonite content of SA composite, while MB sorption decreased.

Uyar and co-workers [82] demonstrated that the drying method of composite beads could have a significant influence onto subsequent sorption of different types of pollutants. Composites that were deep-freezed at −21 °C presented a drastically modified morphology of beads and improved surface area and sorption capacity, compared with beads dried at room temperature. The pollutants sorption capacity and selectivity of synthesized composite beads could be drastically improved by subsequent grafting of different small molecules [98] or polymers (PEI [55], polyaniline [61]) onto the solid beads surface. The PEI beads modified with 3-chloropropanesulfonyl chloride exhibited high removal percentage for Hg^2+^ (>87%) and high selectivity in the presence of competing ions (Mn^2+^, Ni^2+^, Fe^2+^, Pb^2+^, Zn^2+^, and Cr^3+^) [98]. The grafting of PEI onto CS beads, gelated in basic media and sodium dodecylsulphate, increased ~3 times the sorption capacity of composite beads toward RB5 [55]. Also, the grafting of polyaniline nanofibers onto SA beads by a sorption-restricted polymerization method [61] increased the affinity of support for Cu^2+^ and Pb^2+^ in aqueous media. In the majority of all mentioned studies, the pollutant sorption processes in batch experiments [52,56,63] could be modeled by Langmuir or Freundlich isotherms, pseudo-first or pseudo-second order rate, and intra-particle diffusion model, while Thomas isotherm model was more applicable in column studies [59].

### 3.3. Core-Shell Composites

Many scientists conducted pollutant sorption studies on different types of small inorganic (SiO_2_, TiO_2_, Fe_3_O_4_, clays, minerals, etc.) and organic (active carbon, GO, biochar) solid surfaces [126]. The pollutant immobilization on a certain surface strongly depends on the nature, concentration, distribution and accessibility of material functional groups. Inorganic sorbents present very good kinetics but low pollutant sorption capacity relative to sorbent amount. Polyelectrolytes, with high number of functional groups on unit mass, present very high sorption capacities but low kinetics due to slow diffusion in the collapsed state. Therefore, combination of two categories, inorganic solids and polyelectrolytes, can generate new materials with upgraded properties, mechanical stability, and high sorbate accessibility to a high number of functional groups distributed on a high surface area, fast regeneration, and reusability. These composite materials, with a core-shell design, fulfill the utility in pollutant removal by fast, selective, and high amount immobilization on solid phase.

**Figure 7:**
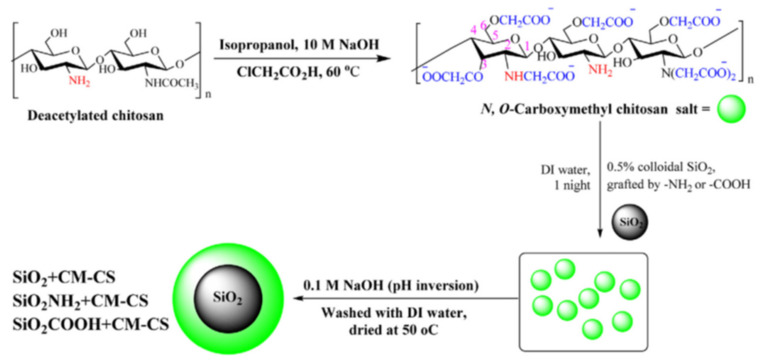
Synthesis of a core-shell composite based on carboxymethylchitosan (CM-CS) and silica particles (reprinted with permission from ref. [83]).

The core-shell architecture could be achieved by direct deposition of different types of natural/synthetic polyelectrolytes (CS, PEI, humic acid, PAH, PVAm, PAA, PSS etc.) on 2D-cores, such as GO [21,22,23,25,115,116] and Ti_3_C_2_-MXenes [117], or 3D-cores, including Fe_3_O_4_ [57,108,122,123,126,127], SiO_2_ [24,28,83,90,100,101,103,109], SiO_2_/Fe_3_O_4_ [88,128], PS [91], FeS [129], clays [99,111,112], CaCO_3_ [102], mesoporous diatomite [89], natural fibers [107] and sand [123]. The direct deposition of the organic or organic/inorganic “shell” onto inorganic or organic “core” could be carried out in (i) one-step, by physisorption [21,89,108,111,112], grafting [23,83,88,100,115,117], ionic or solvent gelation/precipitation [25,27,57,69,128] or (ii) a multi-step procedure, such as layer-by-layer [24,90,101,102,103,109]. Using a strong polyelectrolyte (PSS), Chong and co-workers [108] obtained a stable magnetite nanoparticles with excellent dye removal efficiency (~94%). By simple PSS sorption onto Fe_3_O_4_, the dynamic light scattering and electrophoretic measurements showed a constant hydrodynamic diameter of 150 nm of the magnetic composites over 5 h measuring. This stable dispersion had 50% higher dye sorption capacity compared with bare magnetite nanoparticles. Also, Yao and co-workers [129] obtained stable core-shell colloids (~65–90 nm) based on FeS and PAA with increased sorption properties for Cr^6+^ sorption compared with unmodified FeS. Using direct deposition, by pH inversion precipitation of carboxymethylchitosan onto unmodified and modified silica particles, Aden and collaborators [83] obtained core-shell composites with excellent sorption properties for Ni^2+^ (Figure 7). After drying at 100 °C for 24 h, the composite particles were used without further modification steps as Ni^2+^ ion sorbent at pH 5 and 7.

**Figure 8:**
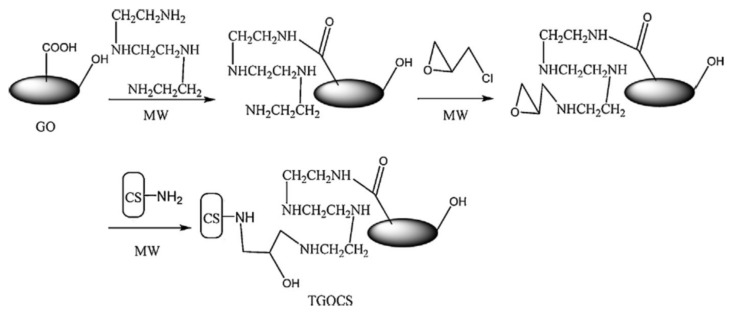
The synthesis of CS/GO composites (reprinted with permission from ref. [23]).

Sometimes, due to harsh environment conditions (extreme pH, high ionic strength, temperature, etc.), which can delaminate the “shell” of composite, the direct grafting of polymeric chains to the solid “core” or “shell” cross-linking after or during deposition, must be carried out. For more stable composites over wide ranges of environment stimuli, many authors anchor polyelectrolyte chains covalently to the inorganic core, which contains a linker molecule on surface [88,100,115]. In this way, the new created core-shell composite is more stable in aqueous media and more effective in sorption of different pollutants. The immobilization of “shell” around the “core” could be achieved by chemical cross-linking with bifunctional compounds, such as glutaraldehyde [89,102,103,109,115], epichlorohydrin [23], α,α’-dichloro-*p*-xylene [24], phtaldialdehyde [91]. Ge and Ma [23] obtained CS/GO composite microparticles by microwave irradiation method using GO, triethylenetetraamine, ECH and CS (Figure 8). Sorption of Cr(VI) onto composite particles showed high values, reaching 219 mg/g at pH 2. The results obtained by batch experiments showed that sorption capacity of synthesized composite increased with temperature and the sorbent material could be recyclable.

**Figure 9:**
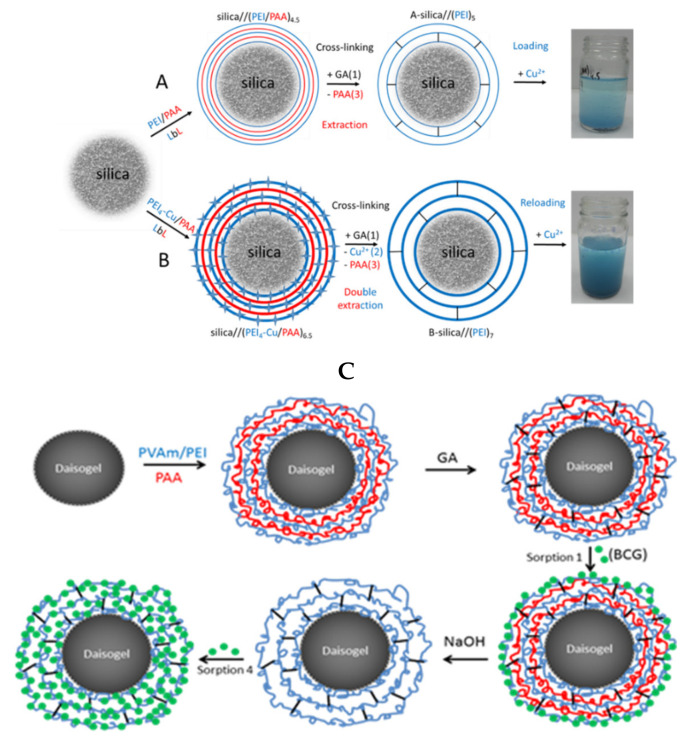
Schematic representation of LbL strategies to obtain silica//(polyelectrolyte)_n_ with high capacities in removal of Cu^2+^ (**A**,**B**) (reprinted with permission from ref. [101]) and dyes (**C**) (reprinted with permission from ref. [109]) from aqueous systems.

Bucatariu and co-workers used selective cross-linking to obtain core-shell silica composites based on PEI, PVAm, polylysine, and PAH. This type of composites has been utilized in the removal of dyes [109] and heavy metal ions [90,101,103] from real and simulated waters. The layer-by-layer technique involved in these studies allowed a controlled deposited polyelectrolyte amount onto spherical silica particles. Furthermore, the subsequent glutaraldehyde cross-linking stabilized the polycation layer onto each individual solid particle. To increase the multilayer flexibility and functional groups accessibility toward pollutant molecules, the polyanion has been removed from the cross-linked multilayer in extreme basic medium, as it can be seen in Figure 9. The fast kinetic of Cu^2+^ sorption and high sorbed amount of anionic dyes (BCG, CR) after polyanion extraction, confirmed the polyelectrolyte multilayer stability and flexibility after cross-linking and polyanion extraction. Based on distribution parameter and relative atomic concentration of elements on surface, the authors demonstrated that silica/(PEI)_10_ composite particles can clean (>95%) a simulated water contaminated with four heavy metal ions (Cu^2+^, Co^2+^, Ni^2+^, Cd^2+^), if the ratio between number of composite functional groups and number of ions is higher than ~9 (non-competitive regime). The competitive sorption between different metal ions showed that the composite had high selectivity for Cu^2+^. Subsequent chemical modification of stable cross-linked core-shell composites with small molecules, i.e., disodium ethylenediamine tetraacetate (EDTA-2Na), increased the sorption capacities by creation of new functional groups onto core-shell surface.

Zhang and co-workers [21] obtained GO/CS composites modified with EDTA-2Na, with higher sorption capacity for Cr^6+^ than unmodified GO/CS. The authors showed that ion sorption strongly depends on pH and GO/CS composite could be used in 7 multiple sorption/desorption cycles with a loss capacity of 5%. Removing phosphate and arsenate ions from eutrophic waters is, also, of paramount importance, therefore the design of low-cost, high-efficient composite sorbents for anionic charged pollutants removal has remained a huge challenge. Kloster and co-workers [130] and Pincus and co-workers [131] created nano-iron/chitosan composites for arsen removal from aqueous media, while Zong and co-workers [132] modified lignin with PEI to create an excellent composite for phosphate ions removal. These composites showed a fast and high removal capacity toward the anionic species.

### 3.4. Composite Gels (Hydrogels, Monolith Cryogels, Sponges)

Composite gels based on polyelectrolytes represent a class of composite soft materials consisting of hydrophilic charged/uncharged chains that absorb a high content of water (>95%) [133]. These low cost and high efficient 3D-dimensional networks are based on polyelectrolytes of different types: (i) synthetic, such as: PEI [36,92], PAH [93,118], PAA [38], PAMPS [106,118,119], or (ii) natural, like the following: CS [30,32,33,34,38,39], modified CS [37,84,85], SA [33,70,73], modified SA [72], modified cellulose [104]. Many studies reported the incorporation of organic/inorganic entities into the gel structure, including GO [70,73], Fe_3_O_4_ [36,106], SiO_2_ [113], zeolites [30,32], clays [72]. In this way, these gel composites gain more structural integrity, useful in the water treatment field. The presence in the gel structure of different types of functional groups (-NH_2_, -NH-, -COOH, -SO_3_H, -OH), confirmed by the solid-state characterization methods, facilitates the pollutant sorption from aqueous environment. The gel composite architectures presenting a high number of functional groups are obtained by different polymerization methods, as presented in Table 2. The synthesis and stability of composite gels depends on the polyelectrolyte chain concentration, pH of medium, ionic strength, temperature, nature of ionic groups, which dictates the subsequent types of interactions between chains and polymer mixing ratios. By simple mixing, Ramakrishnan and collaborators [34] obtained CS/karaya gum composite sponges with removal capacity for both anionic (MO) and cationic (MB) dyes.

**Figure 10:**
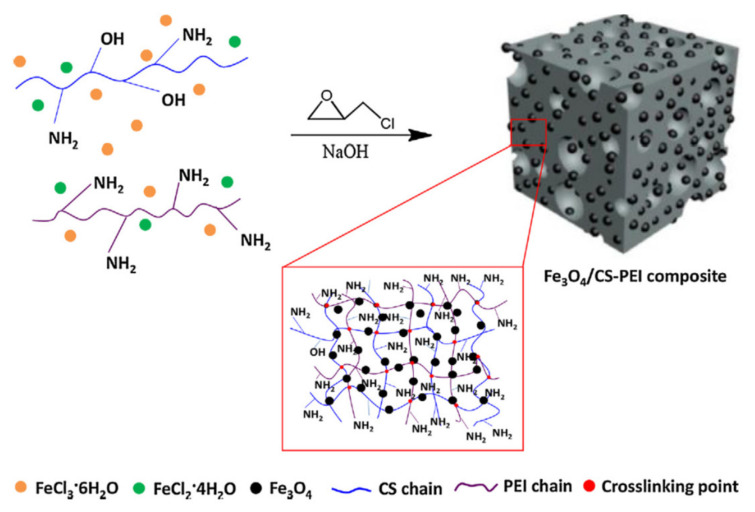
Synthesis of composite gel based on F_3_O_4_ and CS/PEI (reprintedwith permission from ref. [36]).

The combination of Karaya gum and CS polymeric chains brings a stable composite sponge with negative and positive functional groups. The authors studied the swelling profiles and optimized the ratio between these two natural polyelectrolytes for better sorption properties toward negative and positive charged dyes. Harris and McNeil [104] studied a dye sorption onto a locally formed hydrogel between quaternized cellulose and sulfated wood pulp. In this study, the sorption process took place during gel formation. Combining high functionalities of polyelectrolytes and high porous structures of composite gels with magnetism (Fe_3_O_4_) results in a versatile magnetic porous composite gel with superior sorption capability and facile separation [36,106]. Using one-pot synthesis approach, You and co-workers [36] obtained Fe_3_O_4_/CS-PEI composite nanogels with high number of positive charges and magnetic responsiveness (Figure 10).

The synthesized porous sorbent, with good mechanical strength, had a high sorption capacity for CR due to the high surface area resulted from multi-level pore distribution. To remove hydrogel from sorption medium, Ruiz and co-workers [106] synthesized in situ magnetic particles during the free-radical polymerization of modified gelatin in the presence of sodium styrenesulfonate and AMPS.

**Figure 11:**
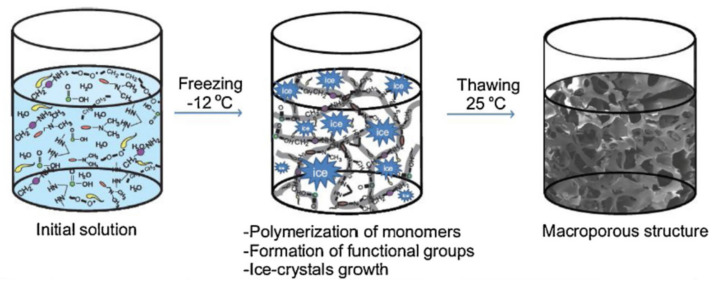
Schematic representation of composite cryogel synthesis (reprinted with permission from ref. [118]).

Magnetic separation is a very promising approach for water treatment due to easy recovery of composite sorbent from contaminated medium by magnetic field. Monoliths with macroporous hydrophilic 3D polymeric matrices could be obtained by free-radical polymerization of different monomeric units at high temperature [124] or subzero temperature of reaction medium [118]. Using an oil/water high internal phase emulsion, Makrygianni and co-workers [124] polymerized the 2-(methacryloxy)ethyl]trimethylammonium chloride in continuous aqueous phase. The authors showed that the gel with 10% cross-linking reached very fast higher water uptake (18 times) and anionic dye subsequent immobilization were characterized by a synergistic of chemisorption and diffusion. 3D monoliths obtained under freezing temperature are denoted cryogels. Also, under freezing temperature of reaction medium, the composite cryogels could be obtained directly from polymers by covalent cross-linking of chains or by monomer polymerization in the presence of other polymer chains or inorganic entities [30,32,84,92,93]. The fast removal and high sorption capacities of cryogels are completed by the modifiability, fast regeneration and high number of reusing cycles of composite cryogels. Baimenov and colaborators [118] obtained macroporous composite cryogels by free-radical polymerization of *N*,*N*-dimethylacrylamide, allylamine, *N*,*N*-methylenebisacrylamide, AMPS) (Figure 11). In this case, the porous structure of gel has been obtained due to ice crystals formed inside, and the 3D network strength is due to the monomer cryocentration effect.

**Figure 12:**
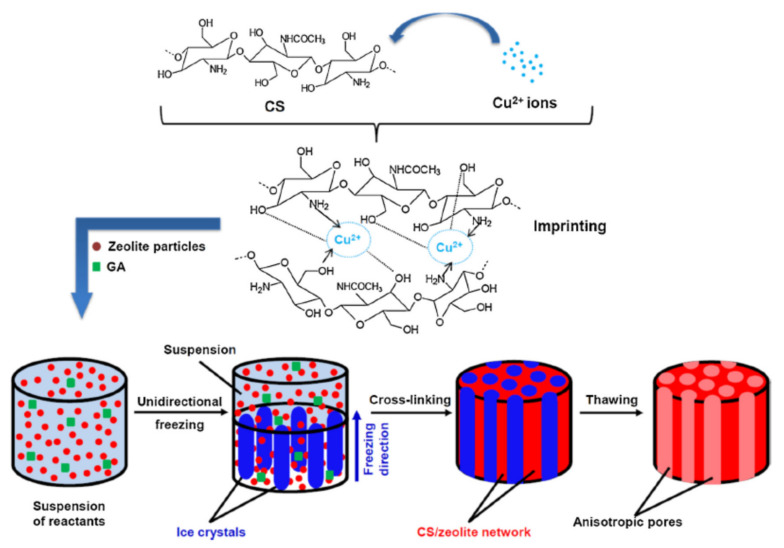
Schematic representation of composite cryogels based on CS and zeolites using a combination of ion-imprinting and freezing methodology (reprintedwith permission from ref. [32]).

The monoliths obtained in pharmaceutical syringes has been sliced in 10 mm diameter pieces, fully characterized, and studied in Hg^2+^ removal from contaminated waters. Humelnicu and co-workers [32] studied the heavy metal ions interactions with a composite cryogel obtained from a zeolite (clinoptilolite) and an ion-imprinting CS in a unidirectional ice-templating method (Figure 12). The CS matrix with zeolite embedded formed an anisotropic composite cryogel where the fast metal ion diffusion has been attributed to the 1D-orientation and interconnectivity of flow channels. The removal efficiency of the synthesized cryogel reached 50% in 30 min. and the equilibrium after 150 min. To study the influence of the imprinting technique, the authors used binary, ternary, and five-component metal ion simulated mixtures. Under competitive conditions, the heavy metal ions followed the sorption order: Cu^2+^ > Fe^3+^ > Ni^2+^ > Zn^2+^ > Cr^3+^.

Cryogels based on metal-chelates with super macroporous structure could had a high affinity towards pharmaceuticals, such as ciprofloxacin [84]. It was shown that cryogel with Cu^2+^ and Al^3+^ inside the carboxyethyl CS network improved the ciprofloxacin recovery up to 98%, if 7 < pH < 10. The sorbent affinity for the drug increased with the metal content, reaching 280 mg and 390 mg/g cryogel for Cu^2+^ and Al^3+^ chelates, respectively.

The composite gels based on polyelectrolytes have been studied by swelling, rheology, morphology, thermal measurements before and after multiple interaction with aqueous pollutants (heavy metals, dyes, pharmaceuticals, pesticides, etc.). Usually, the pollutant molecule immobilization in the gel network is a synergistic process between chemisorption and diffusion, the composite gels being characterized by relative mechanical stability and very high sorbed amounts which make them suitable for using in water treatment technologies.

### 3.5. Nanofibers

**Figure 13:**
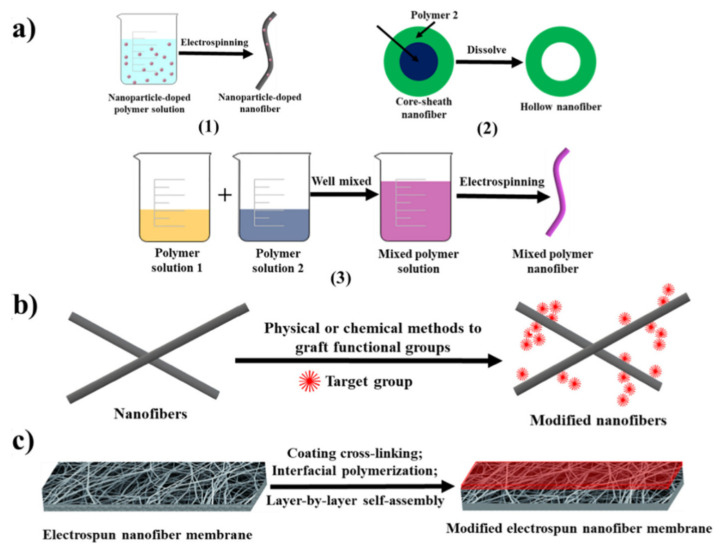
Schematic synthesis of composite nanofibers: (**a**) electrospinning of inorganic/organic polymer mixture, (**b**) surface modification of nanofibers and (**c**) LbL deposited nanofibers (reprinted with permission from ref. [94]).

Increasing the diversity of pollutants in aqueous media requires development of multifunctional membranes, among already discussed composite materials based on polyelectrolytes. A very interesting and practical approach in this direction is the membrane (polymeric, mixed-matrix, ceramic) modifications with composite electrospun nanofibers. The following subsection describes the principal types of polyelectrolytes and other organic/inorganic entities used in nanofibers synthesis, the factors that influence the composite stability and sorption capacities toward different inorganic/organic pollutants. According to Figure 13, composite nanofibers used in pollutant removal by sorption are fabricated by (i) electrospinning of a charged polymer solution or melt in high electric field [13,40,43,44,45,48,49,58,71,74,75,134], (ii) subsequent chemical modification of electrospun nanofibers [46,47,134], and (iii) electrospinning combined with electrospraying techniques [41].

The physico-chemical properties of composite nanofibers depend on the nature of polyelectrolyte and the partner involved, the solvent type, the molecular weight, and concentration of polymeric chains, which determines the viscosity and surface tension of solution. If the polymer concentration is low, the fiber appears as beads on string, and if the concentration is high enough the fibers are homogenous under tensile force [94]. The composite nanofibers with controlled morphology are formed after the solvent evaporation. Composite polyelectrolyte nanofibers based on CS [40,41,43,44,45,46,47,48,49,58], SA [71,74,75], PAA [49,71], and PEI [134] attracted great attention due to high specific surface area, high porosity, superhydrophilicity, and faster sorption rate toward small and large pollutant molecules. To improve solvent resistance and mechanical strength, nanofibers, were annealed at high temperature (150 °C) [49,71], chemically treated with bifunctional agents, e.g., ECH [46], GA [48] etc., or reinforced with organic/inorganic particles, e.g., GO [58], rectorite [41], TiO_2_ [40] etc. Moreover, the reinforcing process increased the sorption capacity of the nanofiber support [40,41,58]. Some authors used poly(vinyl alcohol) to increase the spinnability during the electrospinning process [43,47,58]. Wang and co-workers [71] obtained PAA/SA composite nanofibers by electrospinning followed by a thermal cross-linking at 150 °C. The PAA/SA were characterized by high swelling ratio, excellent stability, high sorption capacity toward Cu^2+^ ions (~600 mg/g nanofiber) and good reusability. Moreover, the authors converted the sorbed toxic Cu^2+^ ions by NaBH_4_ reduction into Cu nanoparticles with subsequent application in catalysis.

**Figure 14:**
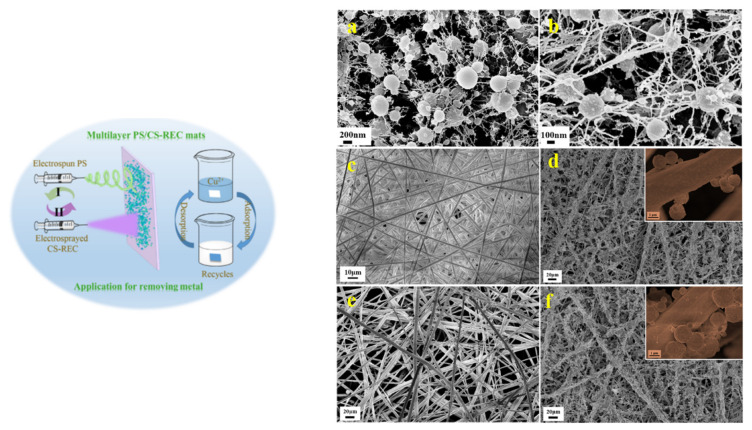
SEM images of nanofibers and composite nanofibers obtained by PS electrospinning alternately with CS-rectorite electrospraying: (**a**) CS, (**b**) CS-rectorite, (**c**) PS, (**d**) PS/CS-rectorite, (**e**) (PS/CS-rectorite)_1.5_ and (**f**) ((PS/CS-rectorite)_2_ (reprinted with permission from ref. [41]).

Chen and collaborators [47] functionalized oxidized CS nanofibers in solid state with an antibacterial agent, polyhexamethylene guanidine. In this study it was shown that this composite, with grafted agent, combined the antibacterial (*S. aureus* and *P. aeruginosa*) activity with very good sorption for Cu^2+^ (57 mg/g) and CR (183 mg/g). Bai and co-workers [58] used GO to synthesize composite nanofibers based on modified CS with a quaternary amine. The authors showed the double role of GO: first, the particles enhanced the ability of polycation to form nanofibers and second, they increased the removal capacity by sorption of porcine parvovirus, a non-enveloped virus. The hydrophobicity of GO and high charge density of polyelectrolyte created a composite with 95% retention for the virus. Razzaz et al. [40] used two methods for CS/TiO_2_ composite nanofibers synthesis: (i) the “coating method”, where electrospun CS nanofibers were dipped in a TiO_2_ dispersion and (ii) “entrapped method”, where composite nanofibers have been obtained by electrospinning of CS and TiO_2_ mixture. The chitosan/TiO_2_ nanofibers prepared by entrapped method have been reused in five consecutive sorption/desorption cycles of Cu^2+^ (710 mg/g) and Pb^2+^ (526 mg/g), the support being more selective for Cu^2+^ in a binary mixture. Composite nanofibers formed from electrospun PS with electrosprayed CS-rectorite (layered silicate) nanospheres on surface, have been synthesized by Tu and co-workers [41]. Also, (PS/CS-rectorite)_n_ composite nanofibers were obtained in a electrospinning-electrospraying alternately deposition approach (Figure 14). In the sorption experiments, the more hydrophylic PS nanofibers coated with CS-rectorite nanospheres showed very good Cu^2+^ sorption (134 mg/g). The authors improved this value by adding Ca^2+^ in the composite, due to additive cation exchange mechanism. After three sorption cycles, the nanofibers maintained the sorption ability at ~75%.

The pollutants removal by the composite nanofibers mentioned above and others, have been carried out in batch experiments [43,47,74] and in dynamic conditions [48]. The adsorption process in batch conditions was successfully modeled by Langmuir [46,74], Freundlich [47,74] or Temkin [47] isotherms, and by pseudo-first or pseudo-second order kinetic models [43,47,75]. The composites nanofibers based on polyelectrolytes have been characterized by modern techniques to obtain information about size and morphology, chemical modifications, distribution of phases, behavior in water etc. The pollutants sorption properties of nanofibers based on polyelectrolytes are strongly related to the nature and ratio of polyelectrolytes and components involved in composite creation, the nanofiber size, which dictates the specific surface area, the swelling of composite, the nature/strength of interactions between support and pollutant and finally, on the characteristic of sorption medium (ionic strength, pH, temperature, solvent, etc.).

### 3.6. Membranes

**Figure 15:**
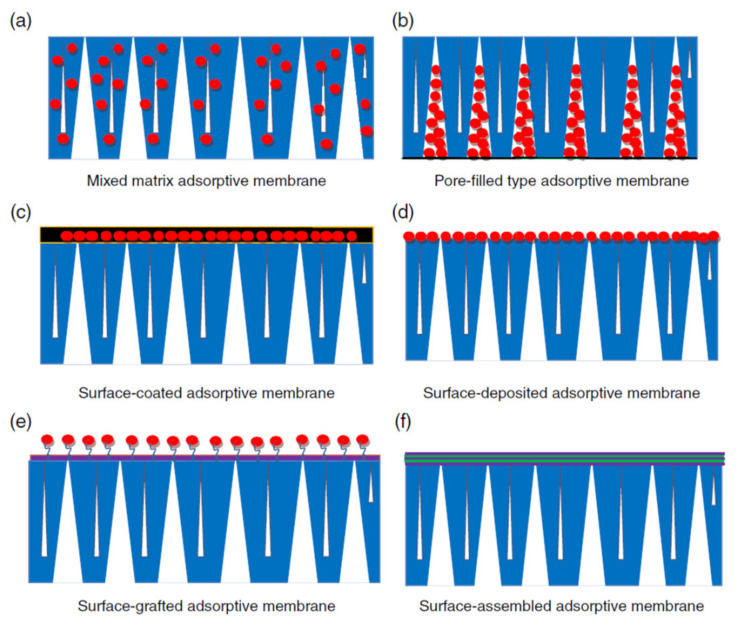
Composite membranes: (**a**) mixed; (**b**) pore-filled; (**c**) surface-coated; (**d**) surface-deposited; (**e**) surface-grafted; (**f**) surface-assembled (reprinted with permission from ref. [142]).

Composite membranes based on polyelectrolytes are one of the most used materials in removal of unwanted molecules from surface/waste waters, due to sorption and rejection functions of it. In this review we took under consideration principal types of composite based on polyelectrolytes which are capable to remove pollutants only by sorptive mechanisms. Because of the large number of reviews published in the field of membranes used in water cleaning [18,135,136,137,138,139,140,141], in this review only some pollutant sorptive composite polyelectrolyte membranes will be exemplified. Sorptive membranes have the same sorption capacity as sorbents, therefore they are known as membrane sorbents (chelating, complexation, and ion exchange membranes) with affinity for heavy metal ions and organic molecules [142]. A schematic overview of the principal types of composite membranes is shown in Figure 15.

The composite membranes can be fabricated by deposition (coating, grafting, self-assembly) of organic/inorganic components onto an unmodified membrane [28,95,143,144,145]. Using a covalent bonded polyelectrolyte onto a cellulose membrane, Pei and collaborators synthesized a sorptive composite membrane with high abilities for metal ions (Cu^2+^, Pb^2+^, Cd^2+^) immobilization. This membrane could be easy regenerated and recovered, reaching a ~200 mg/g sorption capacity. The covalent bonding of a reactive polymer [poly(maleic anhydride-*co*-acrylic acid)] to the cellulose acetate membrane extended the membrane lifetime in water cleaning, and the LbL deposition method increased the polymer amount, therefore increasing the heavy metal sorption capacity [95]. To improve the characteristics of CS membranes, Sahebjamee and co-workers used two methods: (i) a selective dissolution of a porogen [poly(vinyl pyrrolidone)] and (ii) a mixed solvent system with one volatile solvent (acetone) [144]. The authors showed that adding acetone to the composite membrane mixture, the Cu^2+^ sorption capacity increased by 50%. The reusability test with EDTA-2Na demonstrated that after four sorption/desorption cycles, the composite membrane maintained the capacity for heavy metal ions sorption with only 8% reduction.

Due to the low manufacture costs and easy chemistry modification, the composite polymer membranes are popular and frequently used in the water treatment technologies. Such composite membranes still lack some properties required in membrane processes, such as high selectivity towards low solutes. In this way, many studies have been carried out for enhancement of sorption/separation performance of composite membranes. The creation of membranes with high permeability and selectivity, rejection and sorption capacities, antifouling properties are the biggest issues among researchers all over the world who work in the development of membrane materials.

## 4. Conclusions and Future Research Directions

In summary, this study discussed about the multiple synthesis approaches utilized in creation of different types of composite materials based on polyelectrolytes with subsequent application in pollutants removal from synthetic/waste/surface waters.

Throughout this review paper, it has been shown the tremendous potential ability of composite polyelectrolytes in sorption/desorption of inorganic (Cu^2+^, Cd^2+^, Co^2+^, Ni^2+^, Pb^2+^, As^3+/5+^, Fe^2+/3+^, Zn^2+^, Cr^3+/6+^, Hg^2+^, UO_2_^2+^, Mn^2+^, F^−^) and/or organic (dyes, pharmaceuticals, nitrophenol, pesticides, viruses) pollutants. The new materials, created by smartly introducing the inorganic materials in polymeric part or polymer chains onto inorganic part, have overcome the main drawbacks of polyelectrolyte chains, i.e., the “softness” (very low mechanical properties) and the high solubility in water.

In the sorption experiments the physical aspects dictate the pollutant path to the active sorption site from the composite and therefore the first criteria in selection of composite was the composite shape (beads, core-shell, gels, nanofibers, and membranes). Using “soft” polyelectrolytes, with high and diverse number of functional groups, in combination with “hard” inorganic components could results in an “upgraded” composite material with synergic properties in pollutant retention. All pollutants sorption processes could be modeled, mostly by Langmuir and Freundlich isotherms, pseudo-first or pseudo-second order rate. The composites based on polyelectrolytes have been characterized by different solid-state analysis methods, including the following: scanning electron microscopy, Fourier transform infrared spectroscopy, X-ray photoelectron spectrometry, X-ray diffraction, thermogravimetry analysis, dynamic light scattering, transmission electron microscopy, BET analysis. The obtained data provide physico-chemical information about the composite state before and after interaction with different types of pollutants. It can be summarized that composite polyelectrolytes are promising adsorbents for the elimination of inorganic and organic pollutants from wastewater, both in batch and dynamic experimental set-up. There is extensive work done testing these materials using heavy metal ions and dyes as models for the inorganic and organic pollutants, respectively. Only few studies were found to discuss the removal of EPs, such as pharmaceuticals.

The current limitations/challenges of pollutants removal by means of sorption processes using composite polyelectrolytes refer to the following:

- the individual removal of EPs or PPs compounds and not their simultaneous removal, in adsorption columns, the latter being a solution for both the complex nature of wastewater matrix and the operational requirements;
- usually, a combination of two or three AWWT processes might be needed to remove both inorganic and organic PPs/EPs;
- when a combination of sorption is made with processes that are not selective (such as AOPs), intermediates that could be more toxic than the initial compounds could be generated in the final effluent;
- phase transfer of pollutants on solid surfaces (membranes, sorbents) and further regeneration might also contribute to supplementary environmental impacts;
- the technical assessments are used frequently to evaluate the feasibility of sorption or combined processes; there are only few studies that consider the sustainability assessments (by means of Life Cycle Assessment, Life Cycle Costing, carbon footprint, multi-criteria decision analysis, etc.) to design specific functionalized materials.

Overall, this paper could be a starting point in the identification of old “partners/components”, but new by combination methods and different techniques in achieving versatile materials with adaptive behavior for the present demands of wastewater treatment.

## Figures and Tables

**Table 1:** Composite sorbents based on polyelectrolytes used for pollutants removal from aqueous media.

Weak/Strong Polyelectrolytes	Organic/Inorganic Partner	Pollutant Targeted	References
Chitosan (CS)	Poly(ethyleneimine), Poly(vinyl amine), Poly(vinyl alcohol), Poly(*N*,*N*-dimethylamino)ethyl methacrylate, Poly(methacrylic acid), Poly(sodium acrylate), carrageenan, carboxymethyl β-cyclodextrin, carboxyalkyl chitosan, Poly-hexamethylene guanidine, microcrystalline cellulose, sodium alginate, karaya gum, citric acid, itaconic acid, sodium dodecyl sulphate, Sodium lignosulfonate, graphene oxide (GO), Fe_3_O_4_, Fe, TiO_2_, Mesoporous silica structures (MCM-48), silicate rectorite, zeolite, succinic anhydride, maleic anhydride, itaconic acid, trans-aconitic acid, biochar	Congo Red (CR), Methyl Orange (MO), Methylene Blue (MB), Bromocresol Green (BCG), Reactive Black 5 (RB5), Acid blue-113, viruses, Fe(II), Fe(III), Cu(II), Ni(II), Co(II), Cr(III), Cr(VI), Zn(II) As(III), As(V), Cd(II), Pb(II), diclofenac, ciprofloxacin	[13,20,21,22,23,24,25,26,27,28,29,30,31,32,33,34,35,36,37,38,39,40,41,42,43,44,45,46,47,48,49,50,51,52,53,54,55,56,57]
Quaternized chitosan (QCS)	Chitosan, 3-chloro-2-hydroxypropyl trimethyl ammonium chloride, Fe_3_O_4_, GO	MO, CR, Cu(II), Fe(III), Cr(VI)	[20,58,59,60]
Sodium alginate (SA)	Activated carbon, bentonite, activated organo-bentonite, carboxy carbon nanotubes, pillared clay, Mauritanian clay, organo-illite/smectite clay, montmorillonite, nano-hydroxyapatite, carboxymethyl cellulose, microcrystalline cellulose, polyaniline, poly(acrylic acid) glutaraldehyde, Poly(hydroxybutyrate, biochar, CS, GO, Zr(IV), Fe_3_O_4_, MgAl-layered double hydroxide, SiO_2_, aluminum-based metal organic framework and chitosan	Nitrophenol, Pentachlorophenol, polychlorinated biphenyl, crystal violet (CV), MB, MO, As(V), Cu(II), Pb(II), Cd(II), Fe(III), F^-^, Cr(VI), bisphenol A	[14,33,56,59,61,62,63,64,65,66,67,68,69,70,71,72,73,74,75,76,77,78,79,80,81,82]
Carboxyalkyl chitosan (CCS)	CS, salecan, citric acid, Fe_3_O_4_, SiO_2_, Cu(II), Al(III), hexamethylenediisocyanate	As(III), As(V), Ni(II), Pb(II), ciprofloxacin	[52,83,84,85]
Modified Poly(Cyclodextrin)	2,4-toluene diisocyanate, 1,6-hexamethylene diisocyanate, montmorillonite	2,4-dinitrophenol, bisphenol A	[86,87]
Poly(ethyleneimine) (PEI)	Chitosan, Epichlorohydrin (ECH), Poly(acrylic acid) (PAA), poly(vinyl amine), Poly(ethylene glycol) diglycidyl ether, diglycidyl ether of 1,4-butandiol, PS nanoparticles, montmorillonite, cellulose acetate, diatomaceous earth, bacteria, SiO_2_, CaCO_3_, Fe_3_O_4_	Formaldehyde, CR, BCG, Rhodamine B, Hg(II), UO_2_(II), Cd(II), Zn(II), Cu(II), Ni(II), As(III), Mn(II), Cr(III), Cr(VI), Co(II), Fe(II), Pb(II), Zn(II)	[24,36,88,89,90,91,92,93,94,95,96,97,98,99,100,101,102,103]
Carboxymethyl cellulose (CMC)	SA, SiO_2_,	CR, MO, MB, BCG, Pb(II)	[24,79]
Cationic cellulose	Cellulose nanocrystals, wood pulp	MB	[104]
Poly(sodium 4-styrene sulfonate) (PSS)	Octacalcium phosphate, gelatin, pineapple leaf fiber, ZnO, Fe_3_O_4_	Cu(II), Cd(II), tetracycline, CR, MB	[105,106,107,108]
Poly(vinyl amine) (PVAm)	CS, PEI	CR, BCG, Cu(II)	[54,109]
Poly(acrylic acid) (PAA)	PEI, CS, Nano ferrous sulfide, SA	Cd(II), Cr(III), Cr(IV), Cr(VI), Cu(II)	[49,71,97]
Carrageenan	chitosan, hybrid siliceous shells	CR, MB, metoprolol	[31,110]
Anionic polyacrylamide	Kaolinite, montmorillonite, xanthan gum, SiO_2_	Cr(IV), Pb(II), oil	[111,112,113,114]
Poly(allylamine hydrochloride) (PAH)	GO, diglycidyl ether of 1,4-butandiol	Cr(VI), Cu(II), Co(II), Zn(II), Ni(II)	[93,115,116]
Poly(2-acrylamido-2-methylpropane sulfonic acid) (PAMPS)	Ti_3_C_2_-MXenes, methacrylic acid, 2-hydroxyethyl-methacrylate, gelatin, Fe_3_O_4_, CuO with chitosan, alumina	MB, Hg(II), Cu(II), Cd(II), Ni(II), Pb(II), Zn(II), doxycycline, ciprofloxacin	[106,117,118,119,120,121]
Poly{[2-(methacryloyloxy)ethyl]dimethyl-(3-sulfopropyl)ammonium hydroxide-*co*-acrylic acid} (PDMAPS-*co*-AA)	Ti_3_C_2_-MXenes	MB	[117]
Poly(diallyldimethylammonium chloride) (PDDA)	pineapple leaf fiber, ZnO	CR	[107]
Quaternized4-vinyl-pyridine-*co*-acrylamide (QVP-*co*-AAm)	Fe_3_O_4_	CR	[122]
Humic acid (HA)	Fe_3_O_4_	Cu(II)	[123]
Poly{[2-(Methacryloyloxy)ethyl]trimethylammonium chloride}	N,N′ -Methylenebisacrylamide	Orange II, Remazol Brilliant Blue R	[124]

**Table 2:** Synthesis methods of gel composites sorbents.

Synthesis Methods		Monomers or Bifunctional Agents	References
Step 1	Step 2		
Free-radical (co)polymerization	-	Allylamine, N,N-dimethylacrylamide, 2-acrylamido-2-methyl-1-propansulfonic acid (AMPS), methacrylic acid, acrylic acid, 2-hydroxyethylmethacrylate, 2-(methacryloyloxy)ethyl] trimethylammonium chloride, ethyleneglycol dimethacrylate	[38,39,118,119,124]
Mixing of different polymers chains	Ionic gelation	-	[33,73,104]
Mixing of different polymers chains	Covalent cross-linking with bifunctional agents	Glutaraldehyde, hexamethylene diisocyanate, poly(ethyleneglycol) diglycidyl ether, 1,4-butandiol diglycidyl ether, ECH	[30,32,36,84,92,119]
Mixing of polymer chains with monomers	Free-radical polymerization	-	[38,39,106]

## Data Availability

Not applicable.

## Abbreviations

AWWT—advanced wastewater treatment; BCG—Bromocresol Green; CCS—Carboxyalkyl Chitosan; CMC—Carboxymethyl Cellulose; CR—Congo Red; CS—Chitosan; CV—Crystal Violet; ECH—Epichlorohydrin; EDTA-2Na—disodium ethylenediamine tetraacetate; EPs—Emerging pollutants; GO—Graphene Oxide; HA—Humic Acid; HM&D—Heavy metals and dyes; MB—Methylene Blue; MCM-48—mesoporous silica; MO—Methyl Orange; MWWTPs—Municipal wastewater treatment plants; PAA—Poly(acrylic acid); PAH—Poly(allylamine hydrochloride); PDDA—Poly(diallyldimethylammonium chloride); PEI—poly(ethyleneimine); PPs—Priority pollutants; PS—Polystyrene; PSS—Poly(sodium 4-styrene sulfonate); PVAm—poly(vinylamine); QVP-*co*-AAm—Quaternized 4-vinyl-pyridine-*co*-acrylamide; QCS—Quaternized Chitosan; RB5—Reactive Black 5; SA—Sodium Alginate.

## References

[1] Teodosiu C., Gilca A.-F., Barjoveanu G., Fiore S. (2018). Emerging pollutants removal through advanced drinking water treatment: A review on processes and environmental performances assessment. J. Clean. Prod..

[2] Changing How We Produce and Consume: New Circular Economy Action Plan Shows the Way to a Climate-Neutral, Competitive Economy of Empowered Consumers. https://ec.europa.eu/commission/presscorner/detail/en/ip_20_420.

[3] Gherghel A., Teodosiu C., De Gisi S. (2019). A review on wastewater sludge valorisation and its challenges in the context of circular economy. J. Clean. Prod..

[4] Directive 2008/105/CE. https://eur-lex.europa.eu/eli/dir/2008/105/oj.

[5] Decision 2015/495/EU. https://eur-lex.europa.eu/legal-content/EN/TXT/?uri=uriserv%3AOJ.L_.2015.078.01.0040.01.ENG.

[6] Decision 2018/840/EU. https://eur-lex.europa.eu/legal-content/EN/TXT/?uri=CELEX%3A32018D0840.

[7] Bui X.T., Vo T.P.T., Ngo H.H., Guo W.S., Nguyen T.T. (2016). Multicriteria assessment of advanced treatment technologies for micropollutants removal at large-scale applications. Sci. Total Environ..

[8] Shah A.I., Din Dar M.U., Bhat R.A., Singh J.P., Singh K., Bhat S.A. (2020). Prospectives and challenges of wastewater treatment technologies to combat contaminants of emerging concerns. Ecol. Eng..

[9] Yu G., Wang X., Liu J., Jiang P., You S., Ding N., Guo Q., Lin F. (2021). Applications of Nanomaterials for Heavy Metal Removal from Water and Soil: A Review. Sustainability.

[10] Cavallaro G., Micciulla S., Chiappisi L., Lazzara G. (2021). Chitosan-based smart hybrid materials: A physico-chemical perspective. J. Mater. Chem. B.

[11] Mohapi M., Sefadi J.S., Mochane M.J., Magagula S.I., Lebelo K. (2020). Effect of LDHs and Other Clays on Polymer Composite in Adsorptive Removal of Contaminants: A Review. Crystals.

[12] Zheng B., Lin X., Zhang X., Wu D., Matyjaszewski K. (2020). Emerging Functional Porous Polymeric and Carbonaceous Materials for Environmental Treatment and Energy Storage. Adv. Funct. Mater..

[13] Chen S., Yang K., Leng X., Chen M., Novoselov K.S., Andreeva D.V. (2020). Perspectives in the design and application of composites based on graphene derivatives and bio-based polymers. Polym. Int..

[14] Luo J., Huang Z., Liu L., Wang H., Ruan G., Zhao C., Du F. (2021). Recent advances in separation applications of polymerized high internal phase emulsions. J. Sep. Sci..

[15] Abu Elella M.H., Goda E.S., Gab-Allah M.A., Hong S.E., Pandit B., Lee S., Gamal H., Rehman A.U., Yoon K.R. (2021). Xanthan gum-derived materials for applications in environment and eco-friendly materials: A review. J. Environ. Chem. Eng..

[16] Potaufeux J.-E., Odent J., Notta-Cuvier D., Lauro F., Raquez J.-M. (2020). A comprehensive review of the structures and properties of ionic polymeric materials. Polym. Chem..

[17] Wan Ngah W.S., Teong L.C., Hanafiah M.A.K.M. (2011). Adsorption of dyes and heavy metal ions by chitosan composites: A review. Carbohydr. Polym..

[18] Ghiorghita C.-A., Mihai M. (2021). Recent developments in layer-by-layer assembled systems application in water purification. Chemosphere.

[19] Ofridam F., Tarhini M., Lebaz N., Gagnière É., Mangin D., Elaissari A. (2021). pH-sensitive polymers: Classification and some fine potential applications. Polym. Adv. Technol..

[20] Cai L., Ying D., Liang X., Zhu M., Lin X., Xu Q., Cai Z., Xu X., Zhang L. (2021). A novel cationic polyelectrolyte microsphere for ultrafast and ultra-efficient removal of heavy metal ions and dyes. Chem. Eng. J..

[21] Zhang L., Luo H., Liu P., Fang W., Geng J. (2016). A novel modified graphene oxide/chitosan composite used as an adsorbent for Cr(VI) in aqueous solutions. Int. J. Biol. Macromol..

[22] Menazea A.A., Ezzat H.A., Omara W., Basyouni O.H., Ibrahim S.A., Mohamed A.A., Tawfik W., Ibrahim M.A. (2020). Chitosan/graphene oxide composite as an effective removal of Ni, Cu, As, Cd and Pb from wastewater. Comput. Theor. Chem..

[23] Ge H., Ma Z. (2015). Microwave preparation of triethylenetetramine modified graphene oxide/chitosan composite for adsorption of Cr(VI). Carbohydr. Polym..

[24] Ghiorghita C.A., Bucatariu F., Dragan E.S. (2018). Novel silica/polyelectrolyte multilayer core-shell composite microparticles with selectivity for anionic dyes. Cellul. Chem. Technol..

[25] Yan H., Yang H., Li A., Cheng R. (2016). pH-tunable surface charge of chitosan/graphene oxide composite adsorbent for efficient removal of multiple pollutants from water. Chem. Eng. J..

[26] Wang Y., Li L., Luo C., Wang X., Duan H. (2016). Removal of Pb^2+^ from water environment using a novel magnetic chitosan/graphene oxide imprinted Pb ^2+^. Int. J. Biol. Macromol..

[27] Hosain A.N.A., El Nemr A., El Sikaily A., Mahmoud M.E., Amira M.F. (2020). Surface modifications of nanochitosan coated magnetic nanoparticles and their applications in Pb(II), Cu(II) and Cd(II) removal. J. Environ. Chem. Eng..

[28] Jiang Y., Abukhadra M.R., Refay N.M., Sharaf M.F., El-Meligy M.A., Awwad E.M. (2020). Synthesis of chitosan/MCM-48 and β-cyclodextrin/MCM-48 composites as bio-adsorbents for environmental removal of Cd^2+^ ions; kinetic and equilibrium studies. React. Funct. Polym..

[29] Habiba U., Siddique T.A., Joo T.C., Salleh A., Ang B.C., Afifi A.M. (2017). Synthesis of chitosan/polyvinyl alcohol/zeolite composite for removal of methyl orange, Congo red and chromium(VI) by flocculation/adsorption. Carbohydr. Polym..

[30] Humelnicu D., Dragan E.S., Ignat M., Dinu M.V. (2020). A Comparative Study on Cu^2+^, Zn^2+^, Ni^2+^, Fe^3+^, and Cr^3+^ Metal Ions Removal from Industrial Wastewaters by Chitosan-Based Composite Cryogels. Molecules.

[31] Liang X., Duan J., Xu Q., Wei X., Lu A., Zhang L. (2017). Ampholytic microspheres constructed from chitosan and carrageenan in alkali/urea aqueous solution for purification of various wastewater. Chem. Eng. J..

[32] Humelnicu D., Lazar M.M., Ignat M., Dinu I.A., Dragan E.S., Dinu M.V. (2020). Removal of heavy metal ions from multi-component aqueous solutions by eco-friendly and low-cost composite sorbents with anisotropic pores. J. Hazard. Mater..

[33] Tang S., Yang J., Lin L., Peng K., Chen Y., Jin S., Yao W. (2020). Construction of physically crosslinked chitosan/sodium alginate/calcium ion double-network hydrogel and its application to heavy metal ions removal. Chem. Eng. J..

[34] Ramakrishnan R.K., Padil V.V.T., Wacławek S., Černík M., Varma R.S. (2021). Eco-Friendly and Economic, Adsorptive Removal of Cationic and Anionic Dyes by Bio-Based Karaya Gum—Chitosan Sponge. Polymers.

[35] Dinu M.V., Dragan E.S. (2010). Evaluation of Cu^2+^, Co^2+^ and Ni^2+^ ions removal from aqueous solution using a novel chitosan/clinoptilolite composite: Kinetics and isotherms. Chem. Eng. J..

[36] You L., Huang C., Lu F., Wang A., Liu X., Zhang Q. (2018). Facile synthesis of high performance porous magnetic chitosan-polyethylenimine polymer composite for Congo red removal. Int. J. Biol. Macromol..

[37] Ghiorghita C.-A., Borchert K.B.L., Vasiliu A.-L., Zaharia M.-M., Schwarz D., Mihai M. (2020). Porous thiourea-grafted-chitosan hydrogels: Synthesis and sorption of toxic metal ions from contaminated waters. Colloids Surf. A Physicochem. Eng. Asp..

[38] Pan J., Zhu J., Cheng F. (2021). Preparation of Sodium Lignosulfonate/Chitosan Adsorbent and Application of Pb^2+^ Treatment in Water. Sustainability.

[39] Milosavljević N.B., Ristić M.Đ., Perić-Grujić A.A., Filipović J.M., Štrbac S.B., Rakočević Z.L., Krušić M.T.K. (2011). Removal of Cu^2+^ ions using hydrogels of chitosan, itaconic and methacrylic acid: FTIR, SEM/EDX, AFM, kinetic and equilibrium study. Colloids Surf. A Physicochem. Eng. Asp..

[40] Razzaz A., Ghorban S., Hosayni L., Irani M., Aliabadi M. (2016). Chitosan nanofibers functionalized by TiO_2_ nanoparticles for the removal of heavy metal ions. J. Taiwan Inst. Chem. Eng..

[41] Tu H., Huang M., Yi Y., Li Z., Zhan Y., Chen J., Wu Y., Shi X., Deng H., Du Y. (2017). Chitosan-rectorite nanospheres immobilized on polystyrene fibrous mats via alternate electrospinning/electrospraying techniques for copper ions adsorption. Appl. Surf. Sci..

[42] Tajuddin Sikder M., Tanaka S., Saito T., Kurasaki M. (2014). Application of zerovalent iron impregnated chitosan-caboxymethyl-β-cyclodextrin composite beads as arsenic sorbent. J. Environ. Chem. Eng..

[43] Karim M.R., Aijaz M.O., Alharth N.H., Alharbi H.F., Al-Mubaddel F.S., Awual M.R. (2019). Composite nanofibers membranes of poly(vinyl alcohol)/chitosan for selective lead(II) and cadmium(II) ions removal from wastewater. Ecotoxicol. Environ. Saf..

[44] Li L., Li Y., Cao L., Yang C. (2015). Enhanced chromium (VI) adsorption using nanosized chitosan fibers tailored by electrospinning. Carbohydr. Polym..

[45] Li C., Lou T., Yan X., Long Y., Cui G., Wang X. (2018). Fabrication of pure chitosan nanofibrous membranes as effective absorbent for dye removal. Int. J. Biol. Macromol..

[46] Haider S., Ali F.A.A., Haider A., Al-Masry W.A., Al-Zeghayer Y. (2018). Novel route for amine grafting to chitosan electrospun nanofibers membrane for the removal of copper and lead ions from aqueous medium. Carbohydr. Polym..

[47] Chen S., Li C., Hou T., Cai Y., Liang L., Chen L., Li M. (2019). Polyhexamethylene guanidine functionalized chitosan nanofiber membrane with superior adsorption and antibacterial performances. React. Funct. Polym..

[48] Li L., Zhang J., Li Y., Yang C. (2017). Removal of Cr (VI) with a spiral wound chitosan nanofiber membrane module via dead-end filtration. J. Memb. Sci..

[49] Jiang M., Han T., Wang J., Shao L., Qi C., Zhang X.M., Liu C., Liu X. (2018). Removal of heavy metal chromium using cross-linked chitosan composite nanofiber mats. Int. J. Biol. Macromol..

[50] Tzereme A., Christodoulou E., Kyzas G.Z., Kostoglou M., Bikiaris D.N., Lambropoulou D.A. (2019). Chitosan grafted adsorbents for diclofenac pharmaceutical compound removal from single-component aqueous solutions and mixtures. Polymers.

[51] Afzal M.Z., Sun X.-F., Liu J., Song C., Wang S.-G., Javed A. (2018). Enhancement of ciprofloxacin sorption on chitosan/biochar hydrogel beads. Sci. Total Environ..

[52] Mi F.-L., Wu S.-J., Chen Y.-C. (2015). Combination of carboxymethyl chitosan-coated magnetic nanoparticles and chitosan-citrate complex gel beads as a novel magnetic adsorbent. Carbohydr. Polym..

[53] Dragan E.S., Loghin D.F.A. (2018). Fabrication and characterization of composite cryobeads based on chitosan and starches-g-PAN as efficient and reusable biosorbents for removal of Cu^2+^, Ni^2+^, and Co^2+^ ions. Int. J. Biol. Macromol..

[54] Dragan E.S., Cocarta A.I., Dinu M.V. (2014). Facile fabrication of chitosan/poly(vinyl amine) composite beads with enhanced sorption of Cu^2+^. Equilibrium, kinetics, and thermodynamics. Chem. Eng. J..

[55] Chatterjee S., Chatterjee T., Woo S.H. (2011). Influence of the polyethyleneimine grafting on the adsorption capacity of chitosan beads for Reactive Black 5 from aqueous solutions. Chem. Eng. J..

[56] Vijayalakshmi K., Gomathi T., Latha S., Hajeeth T., Sudha P.N. (2016). Removal of copper(II) from aqueous solution using nanochitosan/sodium alginate/microcrystalline cellulose beads. Int. J. Biol. Macromol..

[57] Zhang H., Tan X., Qiu T., Zhou L., Li R., Deng Z. (2019). A novel and biocompatible Fe3O4 loaded chitosan polyelectrolyte nanoparticles for the removal of Cd^2+^ ion. Int. J. Biol. Macromol..

[58] Bai B., Mi X., Xiang X., Heiden P.A., Heldt C.L. (2013). Non-enveloped virus reduction with quaternized chitosan nanofibers containing graphene. Carbohydr. Res..

[59] Li X., Qi Y., Li Y., Zhang Y., He X., Wang Y. (2013). Novel magnetic beads based on sodium alginate gel crosslinked by zirconium(IV) and their effective removal for Pb^2+^ in aqueous solutions by using a batch and continuous systems. Bioresour. Technol..

[60] Ke P., Zeng D., Xu K., Cui J., Li X., Wang G. (2020). Preparation of Quaternary Ammonium Salt-Modified Chitosan Microspheres and Their Application in Dyeing Wastewater Treatment. ACS Omega.

[61] Jiang N., Xu Y., Dai Y., Luo W., Dai L. (2012). Polyaniline nanofibers assembled on alginate microsphere for Cu^2+^ and Pb^2+^ uptake. J. Hazard. Mater..

[62] Lu T., Xiang T., Huang X.-L., Li C., Zhao W.-F., Zhang Q., Zhao C.-S. (2015). Post-crosslinking towards stimuli-responsive sodium alginate beads for the removal of dye and heavy metals. Carbohydr. Polym..

[63] Belhouchat N., Zaghouane-Boudiaf H., Viseras C. (2017). Removal of anionic and cationic dyes from aqueous solution with activated organo-bentonite/sodium alginate encapsulated beads. Appl. Clay Sci..

[64] Sun J., Chen Y., Yu H., Yan L., Du B., Pei Z. (2018). Removal of Cu^2+^, Cd^2+^ and Pb^2+^ from aqueous solutions by magnetic alginate microsphere based on Fe3O4/MgAl-layered double hydroxide. J. Colloid Interface Sci..

[65] Soltani R.D.C., Khorramabadi G.S., Khataee A.R., Jorfi S. (2014). Silica nanopowders/alginate composite for adsorption of lead (II) ions in aqueous solutions. J. Taiwan Inst. Chem. Eng..

[66] Pandi K., Viswanathan N. (2014). Synthesis of alginate bioencapsulated nano-hydroxyapatite composite for selective fluoride sorption. Carbohydr. Polym..

[67] Gopalakannan V., Viswanathan N. (2015). Synthesis of magnetic alginate hybrid beads for efficient chromium (VI) removal. Int. J. Biol. Macromol..

[68] Barreca S., Orecchio S., Pace A. (2014). The effect of montmorillonite clay in alginate gel beads for polychlorinated biphenyl adsorption: Isothermal and kinetic studies. Appl. Clay Sci..

[69] Do X.-H., Lee B.-K. (2013). Removal of Pb^2+^ using a biochar–alginate capsule in aqueous solution and capsule regeneration. J. Environ. Manage..

[70] Zhuang Y., Yu F., Chen J., Ma J. (2016). Batch and column adsorption of methylene blue by graphene/alginate nanocomposite: Comparison of single-network and double-network hydrogels. J. Environ. Chem. Eng..

[71] Wang M., Li X., Zhang T., Deng L., Li P., Wang X., Hsiao B.S. (2018). Eco-friendly poly(acrylic acid)-sodium alginate nanofibrous hydrogel: A multifunctional platform for superior removal of Cu(II) and sustainable catalytic applications. Colloids Surf. A Physicochem. Eng. Asp..

[72] Wang Y., Wang W., Wang A. (2013). Efficient adsorption of methylene blue on an alginate-based nanocomposite hydrogel enhanced by organo-illite/smectite clay. Chem. Eng. J..

[73] Jiao C., Xiong J., Tao J., Xu S., Zhang D., Lin H., Chen Y. (2016). Sodium alginate/graphene oxide aerogel with enhanced strength–toughness and its heavy metal adsorption study. Int. J. Biol. Macromol..

[74] Guo J., Zhang Q., Cai Z., Zhao K. (2016). Preparation and dye filtration property of electrospun polyhydroxybutyrate–calcium alginate/carbon nanotubes composite nanofibrous filtration membrane. Sep. Purif. Technol..

[75] Karthik R., Meenakshi S. (2015). Removal of Cr(VI) ions by adsorption onto sodium alginate-polyaniline nanofibers. Int. J. Biol. Macromol..

[76] Ely A., Baudu M., Kankou M.O.S.O., Basly J.-P. (2011). Copper and nitrophenol removal by low cost alginate/Mauritanian clay composite beads. Chem. Eng. J..

[77] Hassan A.F., Abdel-Mohsen A.M., Elhadidy H. (2014). Adsorption of arsenic by activated carbon, calcium alginate and their composite beads. Int. J. Biol. Macromol..

[78] Tan W.S., Ting A.S.Y. (2014). Alginate-immobilized bentonite clay: Adsorption efficacy and reusability for Cu(II) removal from aqueous solution. Bioresour. Technol..

[79] Ren H., Gao Z., Wu D., Jiang J., Sun Y., Luo C. (2016). Efficient Pb(II) removal using sodium alginate–carboxymethyl cellulose gel beads: Preparation, characterization, and adsorption mechanism. Carbohydr. Polym..

[80] Oladipo A.A., Gazi M. (2014). Enhanced removal of crystal violet by low cost alginate/acid activated bentonite composite beads: Optimization and modelling using non-linear regression technique. J. Water Process Eng..

[81] Lezehari M., Baudu M., Bouras O., Basly J.-P. (2012). Fixed-bed column studies of pentachlorophenol removal by use of alginate-encapsulated pillared clay microbeads. J. Colloid Interface Sci..

[82] Uyar G., Kaygusuz H., Erim F.B. (2016). Methylene blue removal by alginate–clay quasi-cryogel beads. React. Funct. Polym..

[83] Aden M., Ubol R.N., Knorr M., Husson J., Euvrard M. (2017). Efficent removal of nickel(II) salts from aqueous solution using carboxymethylchitosan-coated silica particles as adsorbent. Carbohydr. Polym..

[84] Privar Y., Shashura D., Pestov A., Modin E., Baklykov A., Marinin D., Bratskaya S. (2019). Metal-chelate sorbents based on carboxyalkylchitosans: Ciprofloxacin uptake by Cu(II) and Al(III)-chelated cryogels of N-(2-carboxyethyl)chitosan. Int. J. Biol. Macromol..

[85] Hu X., Yan L., Wang Y., Xu M. (2020). Self-assembly of binary oppositely charged polysaccharides into polyelectrolyte complex hydrogel film for facile and efficient Pb^2+^ removal. Chem. Eng. J..

[86] Anne J.M., Boon Y.H., Saad B., Miskam M., Yusoff M.M., Shahriman M.S., Zain N.N.M., Lim V., Raoov M. (2018). b-Cyclodextrin conjugated bifunctional isocyanate linker polymer for enhanced removal of 2,4-dinitrophenol from environmental waters. R. Soc. Open Sci..

[87] Shabtai I.A., Mishael Y.G. (2018). Polycyclodextrin-Clay Composites: Regenerable Dual-Site Sorbents for Bisphenol A Removal from Treated Wastewater. ACS Appl. Mater. Interfaces.

[88] Pang Y., Zeng G., Tang L., Zhang Y., Liu Y., Lei X., Li Z., Zhang J., Xie G. (2011). PEI-grafted magnetic porous powder for highly effective adsorption of heavy metal ions. Desalination.

[89] Nosrati A., Larsson M., Lindén J.B., Zihao Z., Addai-Mensah J., Nydén M. (2017). Polyethyleneimine functionalized mesoporous diatomite particles for selective copper recovery from aqueous media. Int. J. Miner. Process..

[90] Bucatariu F., Schwarz D., Zaharia M., Steinbach C., Ghiorghita C.-A., Schwarz S., Mihai M. (2020). Nanostructured polymer composites for selective heavy metal ion sorption. Colloids Surf. A Physicochem. Eng. Asp..

[91] Tan Y.Z., Wu D., Lee H.T., Wang H., Honciuc A., Chew J.W. (2017). Synthesis of ligand-carrying polymeric nanoparticles for use in extraction and recovery of metal ions. Colloids Surf. A Physicochem. Eng. Asp..

[92] Privar Y., Malakhova I., Pestov A., Fedorets A., Azarova Y., Schwarz S., Bratskaya S. (2018). Polyethyleneimine cryogels for metal ions sorption. Chem. Eng. J..

[93] Malakhova I., Privar Y., Parotkina Y., Mironenko A., Eliseikina M., Balatskiy D., Golikov A., Bratskaya S. (2020). Rational Design of Polyamine-Based Cryogels for Metal Ion Sorption. Molecules.

[94] Chen H., Huang M., Liu Y., Meng L., Ma M. (2020). Functionalized electrospun nanofiber membranes for water treatment: A review. Sci. Total Environ..

[95] Pei X., Gan L., Tong Z., Gao H., Meng S., Zhang W., Wang P., Chen Y. (2021). Robust cellulose-based composite adsorption membrane for heavy metal removal. J. Hazard. Mater..

[96] Saad D.M., Cukrowska E.M., Tutu H. (2011). Development and application of cross-linked polyethylenimine for trace metal and metalloid removal from mining and industrial wastewaters. Toxicol. Environ. Chem..

[97] Bediako J.K., Choi J.-W., Song M.-H., Lim C.-R., Yun Y.-S. (2021). Self-coagulating polyelectrolyte complexes for target-tunable adsorption and separation of metal ions. J. Hazard. Mater..

[98] Saad D.M.G., Cukrowska E.M., Tutu H. (2012). Sulfonated cross-linked polyethylenimine for selective removal of mercury from aqueous solutions. Toxicol. Environ. Chem..

[99] Zvulunov Y., Ben-Barak-Zelas Z., Fishman A., Radian A. (2019). A self-regenerating clay-polymer-bacteria composite for formaldehyde removal from water. Chem. Eng. J..

[100] Gemeay A.H., El-Halwagy M.E., Elsherbiny A.S., Zaki A.B. (2021). Amine-rich quartz nanoparticles for Cu(II) chelation and their application as an efficient catalyst for oxidative degradation of Rhodamine B dye. Environ. Sci. Pollut. Res..

[101] Bucatariu F., Ghiorghita C.-A., Schwarz D., Boita T., Mihai M. (2019). Layer-by-layer polyelectrolyte architectures with ultra-fast and high loading/release properties for copper ions. Colloids Surf. A Physicochem. Eng. Asp..

[102] Zaharia M.-M., Bucatariu F., Doroftei F., Loghin D.-F., Vasiliu A.-L., Mihai M. (2021). Multifunctional CaCO_3_/polyelectrolyte sorbents for heavy metal ions decontamination of synthetic waters. Colloids Surf. A Physicochem. Eng. Asp..

[103] Bucatariu F., Ghiorghita C.-A., Zaharia M.-M., Schwarz S., Simon F., Mihai M. (2020). Removal and Separation of Heavy Metal Ions from Multicomponent Simulated Waters Using Silica/Polyethyleneimine Composite Microparticles. ACS Appl. Mater. Interfaces.

[104] Harris J.T., McNeil A.J. (2020). Localized hydrogels based on cellulose nanofibers and wood pulp for rapid removal of methylene blue. J. Polym. Sci..

[105] Zhu J., Shu J., Yue X., Su Y. (2020). Hollow and porous octacalcium phosphate superstructures mediated by the polyelectrolyte PSS: A superior removal capacity for heavy metal and antibiotics. J. Mater. Sci..

[106] Ruiz C., Vera M., Rivas B.L., Sánchez S., Urbano B.F. (2020). Magnetic methacrylated gelatin- g -polyelectrolyte for methylene blue sorption. RSC Adv..

[107] Deebansok S., Amornsakchai T., Sae-ear P., Siriphannon P., Smith S.M. (2021). Sphere-like and flake-like ZnO immobilized on pineapple leaf fibers as easy-to-recover photocatalyst for the degradation of congo red. J. Environ. Chem. Eng..

[108] Chong W.H., Ng Q.H., Lim J.K., Yeap S.P., Low S.C. (2020). Study on the enhancement of colloidal stable poly(sodium 4-styrene sulfonate) coated magnetite nanoparticles and regeneration capability for rapid magnetophoretic removal of organic dye. J. Chem. Technol. Biotechnol..

[109] Bucatariu F., Ghiorghita C.-A., Dragan E.S. (2018). Cross-linked multilayer films deposited onto silica microparticles with tunable selectivity for anionic dyes. Colloids Surf. A Physicochem. Eng. Asp..

[110] Soares S.F., Simões T.R., António M., Trindade T., Daniel-da-Silva A.L. (2016). Hybrid nanoadsorbents for the magnetically assisted removal of metoprolol from water. Chem. Eng. J..

[111] Fijałkowska G., Wiśniewska M., Szewczuk-Karpisz K. (2020). Adsorption and electrokinetic studies in kaolinite/anionic polyacrylamide/chromate ions system. Colloids Surf. A Physicochem. Eng. Asp..

[112] Fijałkowska G., Szewczuk-Karpisz K., Wiśniewska M. (2020). Anionic polyacrylamide influence on the lead(II) ion accumulation in soil—The study on montmorillonite. J. Environ. Health Sci. Eng..

[113] Ghorai S., Sarkar A., Raoufi M., Panda A.B., Schönherr H., Pal S. (2014). Enhanced Removal of Methylene Blue and Methyl Violet Dyes from Aqueous Solution Using a Nanocomposite of Hydrolyzed Polyacrylamide Grafted Xanthan Gum and Incorporated Nanosilica. ACS Appl. Mater. Interfaces.

[114] Zhang H., Yang H., Sarsenbekuly B., Zhang M., Jiang H., Kang W., Aidarova S. (2020). The advances of organic chromium based polymer gels and their application in improved oil recovery. Adv. Colloid Interface Sci..

[115] Bao S., Yang W., Wang Y., Yu Y., Sun Y. (2021). Highly efficient and ultrafast removal of Cr(VI) in aqueous solution to ppb level by poly(allylamine hydrochloride) covalently cross-linked amino-modified graphene oxide. J. Hazard. Mater..

[116] Liu H., Li S., Sun D., Chen Y., Zhou Y., Lu T. (2014). Layered graphene nanostructures functionalized with NH2-rich polyelectrolytes through self-assembly: Construction and their application in trace Cu(ii) detection. J. Mater. Chem. B.

[117] Lim S., Park H., Kim J.H., Yang J., Kwak C., Kim J., Ryu S.Y., Lee J. (2020). Polyelectrolyte-grafted Ti 3 C 2 -MXenes stable in extreme salinity aquatic conditions for remediation of contaminated subsurface environments. RSC Adv..

[118] Baimenov A.Z., Berillo D.A., Moustakas K., Inglezakis V.J. (2020). Efficient removal of mercury (II) from water by use of cryogels and comparison to commercial adsorbents under environmentally relevant conditions. J. Hazard. Mater..

[119] Elgueta E., Rivas B.L., Mancisidor A., Núñez D., Dahrouch M. (2019). Hydrogels derived from 2-hydroxyethyl-methacrylate and 2-acrylamido-2-methyl-1-propanesulfonic acid, with ability to remove metal cations from wastewater. Polym. Bull..

[120] Rahman N., Varshney P. (2021). Effective removal of doxycycline from aqueous solution using CuO nanoparticles decorated poly(2-acrylamido-2-methyl-1-propanesulfonic acid)/chitosan. Environ. Sci. Pollut. Res..

[121] Dao T.H., Vu T.Q.M., Nguyen N.T., Pham T.T., Nguyen T.L., Yusa S.I., Pham T.D. (2020). Adsorption characteristics of synthesized polyelectrolytes onto alumina nanoparticles and their application in antibiotic removal. Langmuir.

[122] Atta A.M., Ezzat A.O., Moustafa Y.M., Sabeela N.I., Tawfeek A.M., Al-Lohedan H.A., Hashem A.I. (2019). Synthesis of New Magnetic Crosslinked Poly (Ionic Liquid) Nanocomposites for Fast Congo Red Removal from Industrial Wastewater. Nanomaterials.

[123] Lin S., Shi M., Wang Q., Yang J., Zhang G., Liu X., Fan W. (2021). Transport of Cu^2+^ in Unsaturated Porous Medium with Humic Acid/Iron Oxide Nanoparticle (Fe3O4) Amendment. Water.

[124] Makrygianni M., Christofili A., Deimede V. (2021). Emulsion-templated macroporous ammonium based polymers: Synthesis and dye adsorption study. Colloids Surf. A Physicochem. Eng. Asp..

[125] Beaugeard V., Muller J., Graillot A., Ding X., Robin J.-J., Monge S. (2020). Acidic polymeric sorbents for the removal of metallic pollution in water: A review. React. Funct. Polym..

[126] Nnadozie E.C., Ajibade P.A. (2020). Multifunctional Magnetic Oxide Nanoparticle (MNP) Core-Shell: Review of Synthesis, Structural Studies and Application for Wastewater Treatment. Molecules.

[127] Yang F., Du Q., Sui L., Cheng K. (2021). One-step fabrication of artificial humic acid-functionalized colloid-like magnetic biochar for rapid heavy metal removal. Bioresour. Technol..

[128] Li K., Li P., Cai J., Xiao S., Yang H., Li A. (2016). Efficient adsorption of both methyl orange and chromium from their aqueous mixtures using a quaternary ammonium salt modified chitosan magnetic composite adsorbent. Chemosphere.

[129] Yao Y., Mi N., He C., Zhang Y., Yin L., Li J., Wang W., Yang S., He H., Li S. (2020). A novel colloid composited with polyacrylate and nano ferrous sulfide and its efficiency and mechanism of removal of Cr(VI) from Water. J. Hazard. Mater..

[130] Kloster G.A., Valiente M., Marcovich N.E., Mosiewicki M.A. (2020). Adsorption of arsenic onto films based on chitosan and chitosan/nano-iron oxide. Int. J. Biol. Macromol..

[131] Pincus L.N., Petrović P.V., Gonzalez I.S., Stavitski E., Fishman Z.S., Rudel H.E., Anastas P.T., Zimmerman J.B. (2021). Selective adsorption of arsenic over phosphate by transition metal cross-linked chitosan. Chem. Eng. J..

[132] Zong E., Huang G., Liu X., Lei W., Jiang S., Ma Z., Wang J., Song P. (2018). A lignin-based nano-adsorbent for superfast and highly selective removal of phosphate. J. Mater. Chem. A.

[133] Vázquez-González M., Willner I. (2020). Stimuli-Responsive Biomolecule-Based Hydrogels and Their Applications. Angew. Chemie Int. Ed..

[134] Ju X., Lu J.-P., Zhao L.-L., Lu T.-D., Cao X.-L., Jia T.-Z., Wang Y.-C., Sun S.-P. (2021). Electrospun transition layer that enhances the structure and performance of thin-film nanofibrous composite membranes. J. Memb. Sci..

[135] Buruga K., Song H., Shang J., Bolan N., Jagannathan T.K., Kim K.-H. (2019). A review on functional polymer-clay based nanocomposite membranes for treatment of water. J. Hazard. Mater..

[136] Ndiaye I., Vaudreuil S., Bounahmidi T. (2021). Forward Osmosis Process: State-Of-The-Art of Membranes. Sep. Purif. Rev..

[137] Molinari R., Lavorato C., Argurio P. (2020). Application of Hybrid Membrane Processes Coupling Separation and Biological or Chemical Reaction in Advanced Wastewater Treatment. Membranes.

[138] Ma T., Janot J., Balme S. (2020). Track-Etched Nanopore/Membrane: From Fundamental to Applications. Small Methods.

[139] Alkhouzaam A., Qiblawey H. (2021). Functional GO-based membranes for water treatment and desalination: Fabrication methods, performance and advantages. A review. Chemosphere.

[140] Castro-Muñoz R., González-Melgoza L.L., García-Depraect O. (2021). Ongoing progress on novel nanocomposite membranes for the separation of heavy metals from contaminated water. Chemosphere.

[141] Deka P., Verma V.K., Yurembam B., Neog A.B., Raidongia K., Subbiah S. (2021). Performance evaluation of reduced graphene oxide membrane doped with polystyrene sulfonic acid for forward osmosis process. Sustain. Energy Technol. Assess..

[142] Huang Z., Cheng Z. (2020). Recent advances in adsorptive membranes for removal of harmful cations. J. Appl. Polym. Sci..

[143] Wang Z., Kong D., Qiao N., Wang N., Wang Q., Liu H., Zhou Z., Ren Z. (2018). Facile preparation of novel layer-by-layer surface ion-imprinted composite membrane for separation of Cu^2+^ from aqueous solution. Appl. Surf. Sci..

[144] Sahebjamee N., Soltanieh M., Mousavi S.M., Heydarinasab A. (2020). Preparation and characterization of porous chitosan–based membrane with enhanced copper ion adsorption performance. React. Funct. Polym..

[145] Jiang Y., Wang W.-C. (2011). Functional membranes prepared by layer-by-layer assembly and its metal ions adsorption property. Polym. Adv. Technol..

